# *De novo* Assembly and Comparative Analyses of Mitochondrial Genomes in Piperales

**DOI:** 10.1093/gbe/evad041

**Published:** 2023-03-10

**Authors:** Runxian Yu, Xudong Chen, Lingjie Long, Matthias Jost, Ran Zhao, Lumei Liu, Jeffrey P Mower, Claude W dePamphilis, Stefan Wanke, Yuannian Jiao

**Affiliations:** State Key Laboratory of Systematic and Evolutionary Botany, Institute of Botany, The Chinese Academy of Sciences, Beijing, China; College of Life Science, University of Chinese Academy of Sciences, Beijing, China; State Key Laboratory of Systematic and Evolutionary Botany, Institute of Botany, The Chinese Academy of Sciences, Beijing, China; College of Life Science, University of Chinese Academy of Sciences, Beijing, China; State Key Laboratory of Systematic and Evolutionary Botany, Institute of Botany, The Chinese Academy of Sciences, Beijing, China; College of Life Science, University of Chinese Academy of Sciences, Beijing, China; Institute of Botany, Dresden University of Technology, Dresden, Germany; State Key Laboratory of Systematic and Evolutionary Botany, Institute of Botany, The Chinese Academy of Sciences, Beijing, China; State Key Laboratory of Systematic and Evolutionary Botany, Institute of Botany, The Chinese Academy of Sciences, Beijing, China; College of Life Science, University of Chinese Academy of Sciences, Beijing, China; Center for Plant Science Innovation and Department of Agronomy and Horticulture, University of Nebraska, Lincoln, Nebraska; Department of Biology and Huck Institutes of the Life Sciences, The Pennsylvania State University, University Park, Pennsylvania; Institute of Botany, Dresden University of Technology, Dresden, Germany; Departamento de Botanica, Instituto de Biología, Universidad Nacional Autonoma de Mexico, Coyoacan, Distrito Federal, Mexico; State Key Laboratory of Systematic and Evolutionary Botany, Institute of Botany, The Chinese Academy of Sciences, Beijing, China; College of Life Science, University of Chinese Academy of Sciences, Beijing, China

**Keywords:** Piperales, Aristolochiaceae, mitochondrial genome, mutation spectrum, HGT

## Abstract

The mitochondrial genome of *Liriodendron tulipifera* exhibits many ancestral angiosperm features and a remarkably slow evolutionary rate, while mitochondrial genomes of other magnoliids remain yet to be characterized. We assembled nine new mitochondrial genomes, representing all genera of perianth-bearing Piperales, as well as for a member of the sister clade: three complete or nearly complete mitochondrial genomes from Aristolochiaceae and six additional draft assemblies including *Thottea*, Asaraceae, Lactoridaceae, and Hydnoraceae. For comparative purpose, a complete mitochondrial genome was assembled for *Saururus*, a member of the perianth-less Piperales. The average number of short repeats (50–99 bp) was much larger in genus *Aristolochia* than in other angiosperm mitochondrial genomes, and approximately 30% of repeats (<350 bp) were found to have the capacity to mediate recombination. We found mitochondrial genomes in perianth-bearing Piperales comprising conserved repertories of protein-coding genes and rRNAs but variable copy numbers of tRNA genes. We identified several shifts from *cis*- to *trans*-splicing of the Group II introns of nad1i728, cox2i373, and nad7i209. Two short regions of the *cox*1 and *atp*8 genes were likely derived from independent horizontal gene transfer events in perianth-bearing Piperales. We found biased enrichment of specific substitution types in different lineages of magnoliids and the Aristolochiaceae family showed the highest ratio of A:T > T:A substitutions of all other investigated angiosperm groups. Our study reports the first mitochondrial genomes for Piperales and uses this new information for a better understanding of the evolutionary patterns of magnoliids and angiosperms in general.

**Significance**

Magnoliids are one of the major mesangiosperm lineages, besides eudicots, and monocots. Although plastid and nuclear genomes of magnoliids have been deciphered, their mitochondrial genomes, except for *Liriodendron* and *Magnolia* in Magnoliales, remain largely uncharacterized. By assembling new mitochondrial genomes and performing corresponding comparative analyses, we found their features exhibit great genomic diversity in aspects of short repeats, tRNAs, and mutation patterns that are dissimilar to those previously identified in *Liriodendron*. Our results demonstrated that mitochondrial genomes of magnoliids have great diversity and that *Liriodendron*, but not all magnoliids, exhibits a remarkably slow evolutionary rate. This study generated valuable resources of the first mitochondrial genomes in Piperales and provided better understanding of angiosperm mitochondrial genomes broadly.

## Introduction

Mitochondria and plastids are key organelles that play important roles in plants. Their genomes have been extensively altered throughout the long history of co-evolution with the nuclear genome (Johnston 2019). In land plants, the plastid genome often possesses a typical quadripartite structure and conserved genomic content, whereas mitochondrial genomes, especially those of angiosperms, have undergone extensive modification and diversification during the course of evolutionary history (Mower et al. 2012; Mower and Vickrey 2018). Angiosperm mitochondrial genomes are typically ∼300–800 kb in size and assembled in master circles (Mower et al. 2012; Sloan 2013), although the size can vary greatly between or even within species (Wu and Sloan 2019; Lin et al. 2022; Sun et al. 2022). These differences in size are mainly influenced by the proliferation of repetitive elements in intergenic regions (Gandini et al. 2019; Wynn and Christensen 2019), and by insertions of DNA from the plastid and nuclear genomes (Mower et al. 2012; Sloan and Wu 2014). Moreover, the presence of large or intermediately sized repeat elements can mediate recombination, leading to rearranged mitochondrial genomic structures and potential shifting from *cis*- to *trans*-splicing of mitochondrial introns (Qiu and Palmer 2004; Alverson et al. 2011; Guo et al. 2020; Yu et al. 2021). The presence of recombinogenic repeats and foreign DNA makes it challenging to assemble mitochondrial genomes, which hinders the study of their evolution.

In angiosperms, mitochondrial genomes usually contain 25–41 protein-coding genes (PCGs), three ribosomal RNA (rRNA) genes, and nine to 26 transfer RNA (tRNA) genes. Fluctuations in the numbers of these genes are primarily attributed to variations in ribosomal PCGs and plastid-derived tRNA genes (Mower 2020; Warren and Sloan 2020). It has been widely reported that foreign DNA can be integrated into mitochondrial genomes via horizontal gene transfer (HGT), but the volume of foreign DNA may vary greatly between angiosperm lineages (Mower et al. 2010; Davis and Xi 2015; Park et al. 2015; Roulet et al. 2020). A Group I intron in *cox*1 (cox1i728) has been identified as a product of HGT from an unknown fungal donor in multiple unrelated angiosperm species, suggesting great mobility and multiple independent gains of such introns (Cho et al. 1998; Sanchez-Puerta et al. 2008).

The magnoliid clade, as one of the major mesangiosperm lineages, comprises four orders (Magnoliales, Canellales, Laurales, and Piperales) with great species and genetic diversity (Peréz-Mesa et al. 2019; Yang et al. 2020; Suárez-Baron et al. 2021). Previous studies found the mitochondrial genome of *Liriodendron tulipifera* (Magnoliales) exhibits ancestral traits of angiosperms with ancestral gene content and order, abundant editing sites, and remarkably low nucleotide substitution rate (Richardson et al. 2013; Dong et al. 2020). However, it remains unclear whether other magnoliids also exhibit such features.

The Piperales can be separated into a perianth-bearing and a perianth-less clade. The perianth-bearing clade consists of the monophyletic families Aristolochiaceae, Asaraceae, Hydnoraceae, and Lactoridaceae (Jost et al. 2021), which are well-known for their medicinal value and for harboring a high content of carcinogenic chemical compounds, aristolochic acids (AAs) (Han et al. 2019). In perianth-bearing Piperales, previous studies have primarily explored growth-form evolution and interaction with other organisms in a phylogenetic context (Wagner et al. 2014; Allio et al. 2021; Jost et al. 2021). The nuclear genomes of *Aristolochia fimbriata* and *A. contorta* have been fully sequenced and assembled *de novo* to the chromosome level (Qin et al. 2021; Cui et al. 2022). These two genomes lack evidence of lineage-specific whole genome duplications since the origin of extant angiosperms; thus, the genomes largely reflect those of ancestral angiosperms. However, there has been no complete mitochondrial genome assembled and analyzed for the whole order.

To better understand the evolution of perianth-bearing Piperales, it is critical to fully understand their genetic basis, including their nuclear, plastid, and mitochondrial genomes. Here, we assembled mitochondrial genomes for representative perianth-bearing Piperales species covering all genera and explored multiple aspects of genome evolution. Specifically, a total of eight mitochondrial genomes from four autotrophic and two holoparasitic genera were assembled for the first time, and these sequences were compared with mitochondrial genomes from a newly assembled perianth-less Piperales species and other available angiosperms. The genome sizes, gene content, foreign DNA regions, and mutation patterns were investigated.

## Results and Discussion

### De novo Assembly of Piperales Mitochondrial Genomes

We sampled species from all genera of perianth-bearing Piperales. For Aristolochiaceae, *Thottea hainanensis* and three *Aristolochia* species (*A. fimbriata*, *A. gigantea*, and *A. manshuriensis*) were included. These latter species represented two out of three *Aristolochia* subgenera; *A. manshuriensis* belongs to the *Aristolochia* subgenus *Siphisia*, whereas the other two belong to subgenus *Aristolochia*. Both Hydnoraceae genera were represented with the inclusion of *Hydnora visseri* and *Prosopanche americana*. Both Asaraceae genera were also represented by *Asarum sieboldii* and *Saruma henryi*. Lactoridaceae was represented by *Lactoris fernandeziana*. In addition, we sampled *Saururus chinensis* as a representative of perianth-less Piperales.

Illumina DNA-seq generated 11.3 Gb of data each for *A. fimbriata*, *A. gigantea*, *A. manshuriensis*, *Thottea*, *Asarum*, and *Saruma*. By combining Illumina short reads and Nanopore long reads, we successfully assembled the complete mitochondrial genomes of both *Saururus* and *A. fimbriata*; both were arranged into three circular contigs with length of 580,630 and 349,852 bp, respectively (table 1). Using the *A. fimbriata* mitochondrial genome as a reference, we assembled mitochondrial DNA (mtDNA) reads into a single linear contig (328,934 bp) for *A. gigantea*, and five circular contigs (contig1–5) plus one linear contig (contig6 with 71,501 bp in length) for *A. manshuriensis* with a total length of 509,313 bp. Due to the consistency of the DNA-seq coverage depth (supplementary fig. S1, Supplementary Material online), we considered the mitochondrial genomes of *A. gigantea* and *A. manshuriensis* to be nearly complete assemblies. The five additional, newly assembled mitochondrial genomes had five to 44 linear contigs each with a total assembled length ranging from 426,953 to 1,916,195 bp, and were classified as draft assemblies (detailed information see table 1). Most perianth-bearing Piperales mitochondrial genomes were of modest size, ranging from 328,934 to 576,500 bp; those of *Thottea* and *Hydnora* were two-fold and four-fold larger than the others at 1,227,888 bp and 1,916,195 bp, respectively (table 1). Despite great variation in mitochondrial genome size, GC content was consistent (46.4–48.0%) (table 1), demonstrating mtDNA similarity between these species and other angiosperms (Mower et al. 2012). Mitochondrial plastid insertions (MTPT) accounted for a variable but generally small proportion (0.22–11.7%) of each of the studied mitochondrial genomes, with the lowest proportions in the parasitic Hydnoraceae (table 1).

**Table 1:** Summary of the Nine Newly Assembled Mitochondrial Genomes

Feature	*Saururus chinensis*	*Saruma henryi*	*Asarum sieboldii*	*Hydnora visseri*	*Prosopanche americana*	*Thottea hainanensis*	*Aristolochia manshuriensis*	*A. gigantea*	*A. fimbriata*
Assembly version	Complete	Draft	Draft	Draft	Draft	Draft	Nearly complete	Nearly complete	Complete
Assembled contig(s)	3 circular	8 linear	5 linear	16 linear	44 linear	22 linear	5 circular and 1 linear	1 linear	3 circular
Genome size (bp)	580,630	538,686	576,500	1,916,195	426,953	1,227,888	509,313	328,934	349,852
GC content (%)	46.4	46.7	46.9	46.6	46.9	47.2	46.7	48.0	47.9
Repetitive content (%)	15.7	24.3	7.8	28.5	8.6	7.9	4.9	15.1	11.9
MTPT content (%)	3.0	6.6	2.2	0.51	0.22	7.6	11.7	2.0	1.4
Mitochondrial protein-coding gene^a^	39 (40)	41 (43)	41	40 (44)	39	41	41	41	41
rRNA gene^a^	3 (4)	3 (4)	3 (4)	3 (4)	3	3	3	3 (4)	3 (4)
tRNA gene^a^	19 (34)	25 (32)	22 (24)	5	10 (12)	29 (33)	27	20	21
Proportion of protein-coding region (%)	5.9	6.4	6.0	1.9	7.4	2.9	6.8	10.5	9.9

a Total gene copy number (including duplicates) is shown in parentheses.

### Repeats and Structural Variation Among *Aristolochia* Mitochondrial Genomes

**Fig. 1.**
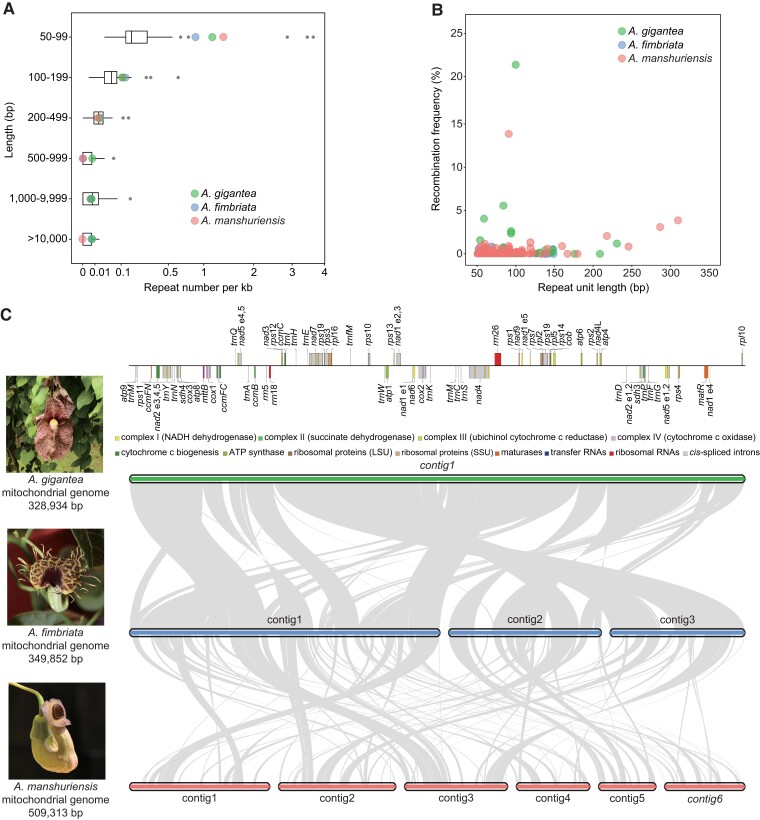
Mitochondrial genome features of three *Aristolochia* species. (*A*) Length distribution of repeats in the complete mitochondrial genomes of 74 angiosperms. Black vertical line: median; box: upper, and lower quartile, including 50% of the distribution; whiskers: minimum and maximum of the data, provided that their length does not exceed 1.5 × the interquartile range; black circles: outliers. (*B*) Recombination frequency for repeats of 50–350 bp in length. (*C*) Synteny among the three *Aristolochia* mitochondrial genomes studied here. Bars and ribbons represent contigs and syntenic regions, respectively (>250 bp). Contig names in standard and italic font represent those with circular and linear structures, respectively. Left, representative flower images and the estimated mitochondrial genome size. Top, annotation map of *Aristolochia gigantea* for reference.

We identified interspersed repeats, which accounted for 3.0% of the *Saururus* mitochondrial genome and 4.9–28.5% of those of perianth-bearing members of Piperales (table 1). Because the *Aristolochia* mitochondrial genomes were complete or nearly so, we focused analyses of repeats and rearrangements on these species. Specifically, 302, 354, and 698 repeats comprising 105, 119, and 244 repeat units were identified in the *A. fimbriata*, *A. gigantea*, and *A. manshuriensis* mitochondrial genomes, respectively. The average number of short repeats (50–99 bp) was significantly larger in *Aristolochia* than in other angiosperm mitochondrial genomes (Wilcoxon rank sum test, *P* value < 0.01); there were 0.87–1.4 repeats per kb in *Aristolochia* compared with only 0.032–0.76 repeats per kb in the majority of other angiosperms (fig. 1*A* and supplementary table S1, Supplementary Material online). Additionally, there were many Illumina DNA-seq reads mapped to alternative conformations. Approximately 30% of repeats (<350 bp) were found to have the capacity to mediate recombination, leading to a putatively highly dynamic mitogenomic structure (fig. 1*B* and supplementary tables S2–S4, Supplementary Material online). In general, mitochondrial short repeats (50–99 bp) in other seed plants are often found to have low recombination activities (Guo et al. 2016; Dong et al. 2018; Yu et al. 2021). The previously known exceptions are those in *Viscum scurruloideum* and *Picea abies* (Skippington et al. 2015; Sullivan et al. 2020).

To further investigate structural variations in mtDNA, we selected three *Aristolochia* mitochondrial genomes as a study system. Pairwise synteny analyses showed that there were abundant rearrangements and inversions between the three genomes (fig. 1*C*). We identified 65 syntenic blocks (totaling 321,437 bp) between the mtDNA of *A. fimbriata* and *A. gigantea* (both members of the subgenus *Aristolochia*), comprising more than 90% of their mitochondrial genomes. Then, we investigated the content of the remaining nonsyntenic regions between *A. fimbriata* and *A. gigantea,* and found this region lacking any mitochondrial gene. We also specifically checked the sequence of these nonsyntenic regions and found they comprise repeats (15.6%), or showing sequence similarity with mitochondrial genomes of other angiosperms (5.3%) and chloroplast genomes (6.2%) (supplementary table S5, Supplementary Material online). Also part of these sequences cannot identify their source. Overall, the evolving pattern of subgenera *Aristolochia* mitochondrial genomes kept in line with other lineages (Christensen 2013; Wu and Sloan 2019; Lin et al. 2022), in which dispensable sequences such as repetitive elements or foreign sequences were the main drivers of genome structure and size variation between closely related species.

When comparing them to the mtDNA of *A. manshuriensis* (subgenus *Siphisia*), the cumulative syntenic blocks only came up to about ∼40% of the size of the *A. manshuriensis* mitochondrial genome. The largest syntenic block in *A. manshuriensis* was only 9,359 bp, compared to 22,240 bp between the two mitochondrial genomes of subgenus *Aristolochia* (supplementary table S6, Supplementary Material online). Although the *A. manshuriensis* mitochondrial genome was nearly complete, the presence of multiple circular contigs and the intact gene repertoire implied that the relatively fragmented syntenic blocks were not due to incorrect assembly. Our results indicated a high level of genome turnover and shuffling after the divergence of the subgenera *Aristolochia* and *Siphisia*.

### Comparative Analysis of Gene Content of Piperales Mitochondrial Genomes

To better understand the genetic diversity among mitochondrial genomes of Piperales, we performed comparative analyses with respect to gene and intron contents. The mitochondrial genomes of *Saururus* and perianth-bearing Piperales all had complete or nearly complete repertoires of PCGs (39–41, excluding duplicates), a full set of three rRNA genes, and a heterogeneous set of five to 29 different tRNA genes (table 1 and supplementary fig. S2, Supplementary Material online). The large variations in the number of tRNA genes among species in perianth-bearing Piperales appear to be due to massive loss of tRNAs from the Hydnoraceae and to large variations in the number of tRNAs of plastid origin. Several intact or pseudogene copies of plastid PCGs were also detected in these genomes (supplementary fig. S3, Supplementary Material online).

**Fig. 2.**
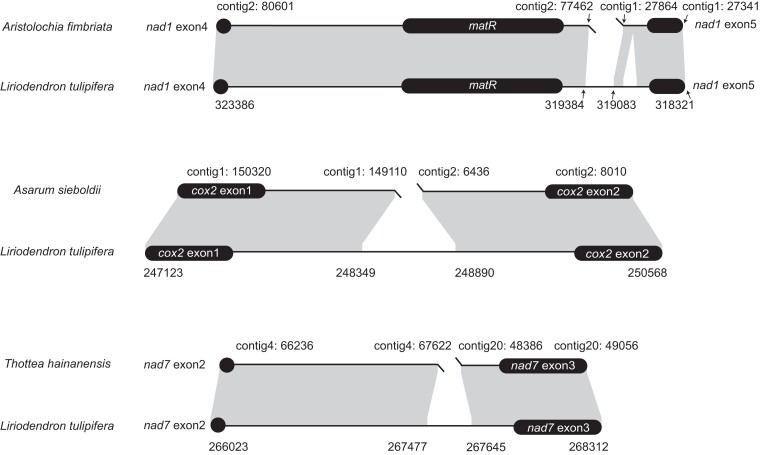
Schematic diagrams showing syntenic analysis of three mitochondrial introns in perianth-bearing Piperales and *Liriodendron tulipifera*. Flanking exons are shown as rounded black rectangles. Shading with genomic coordinates indicates homologous regions between mitochondrial genomes of each pair of species.

Intron content was also variable, including 23–25 Group II introns in each of the newly assembled mitochondrial genomes of perianth-bearing Piperales and one Group I intron (cox1i729) in Hydnoraceae and *Aristolochia* (supplementary table S7, Supplementary Material online). We identified several shifts from *cis*- to *trans*-splicing of Group II introns. This shift in nad1i728 was extensively shared by the mitochondrial genomes of *Saururus* and perianth-bearing Piperales, whereas the splicing shifts in cox2i373 and nad7i209 were specific to *Asarum* and *Thottea*, respectively (fig. 2 and supplementary table S7, Supplementary Material online). Interestingly, although rare, such shifts of cox2i373, nad1i728, and nad7i209 were also identified in other angiosperms and gymnosperms (Kim and Yoon 2010; Guo et al. 2020; Yu et al. 2021), indicating shifts to trans-splicing of mitochondrial *cis*-spliced introns can be the universal phenomena among seed plants.

### HGT in Piperales Mitochondrial Genomes

After integrating orthologous genes of 38 additional diverse angiosperms into our analyses, we constructed a phylogeny for each of the 41 mitochondrial PCGs annotated in *Saururus* and perianth-bearing Piperales to identify potential HGT events. After carefully inspecting the gene trees, we did not find any perianth-bearing Piperales genes clustered with nonmagnoliids with high bootstrap support (>75) (supplementary fig. S4, Supplementary Material online). Furthermore, gene conversion analysis did not indicate the presence of chimeric genes in perianth-bearing Piperales mtDNA; thus, there were no obvious HGT events identified by these analyses.

**Fig. 3.**
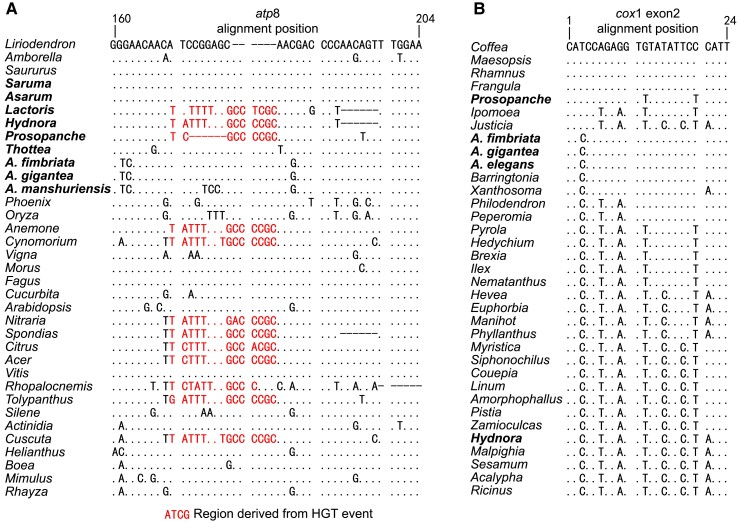
Nucleic acid alignments of *atp*8 and *cox*1. (*A*) Alignment of the *atp*8 gene region containing HGT sequences. (*B*) Alignment of the exon 2 region in *cox*1. Dots and hyphens indicate identical bases and gaps, respectively, compared to the reference sequence. Perianth-bearing Piperales species are shown in bold.

However, after manually checking the alignments in which 48 species were included, we found two short regions in *atp*8 and exon 2 of *cox*1 that showed signs of gene conversion. The *atp*8 conversion was identified only in Lactoridaceae and Hydnoraceae, which is a 15 bp sequence replacing the native 9 bp one. After further inspection of all *atp*8 gene sequences for other species available in GenBank, we found that this short region had counterparts in diverse angiosperms, that is, Ranunculaceae (autotrophic *Anemone*), Sapindales (autotrophic *Acer*, *Citrus*, *Nitraria,* and *Spondias*), and various parasitic lineages (*Cuscuta*, *Cynomorium*, *Rhopalocnemis*, and *Tolypanthus*) (fig. 3*A*). We could not find any inverted repeats flanking the 15 bp region, which excluded the possibility of mobile element derived replacement. Given the high sequence divergence between the 15 bp sequence and the native 9 bp one, it also seems not very likely from independent mutations and could be potentially derived from HGT event. We searched the 15 bp sequence against the National Center for Biotechnology Information (NCBI) NT database (word size = 16; https://blast.ncbi.nlm.nih.gov/Blast.cgi) for potential donor, but no hit was found. More additional sequence data from various species may warrant the identification of the potential donor.

The *cox*1 gene often exhibits one exon in most angiosperms, but an intron (named cox1i729) of *cox*1 was detected in hundreds of angiosperms which was thought to derive from an unknown fungal donor (Cho et al. 1998; Cusimano et al. 2008; Sanchez-Puerta et al. 2008; Sinn and Barrett 2020). The short region at the 5′ end of exon 2 in *cox*1 is well-acknowledged as a co-conversion tract (CCT) associated with the mobile cox1i729 (Cho et al. 1998). Here, we found the cox1i729 intron existing in the studied species of Hydnoraceae and Aristolochia, which was also reported previously (Barkman et al. 2007; Sanchez-Puerta et al. 2017). To understand the history of the intron grain events, we performed phylogenetic inference analysis for cox1i729 in the *Aristolochia* subgenus *Aristolochia* and two parasitic genera (*Hydnora* and *Prosopanche*) and found that the cox1i729 in subgenus *Aristolochia* was sister to two Euphorbiaceae members (*Hevea brasiliensis* and *Manihot esculenta*) with high bootstrap support (82%). In addition, *Hydnora* and *Prosopanche* clustered with *Linum* sp. and *Ipomoea* sp. with low and modest bootstrap support, respectively (supplementary fig. S5, Supplementary Material online). Moreover, we found that the CCTs were identical within *Aristolochia* but diversified in *Hydnora* and *Prosopanche*. Thusly, results from phylogeny and CCT suggested that cox1i729 was independently gained at least three times in perianth-bearing Piperales (fig. 3*B*).

Parasitic plants attach to hosts with a specialized organ, the haustorium, which can function as a “pipe” between organisms (Teixeira-Costa 2021). Haustoria can allow exchange of nutrients and nucleic acids between hosts and parasites (Davis and Xi 2015; Hettenhausen et al. 2017). It has previously been reported that mitochondrial genomes of parasitic plants may possess many foreign DNA fragments from their hosts (Xi et al. 2013; Sanchez-Puerta et al. 2017; Roulet et al. 2020). A previous study detected an HGT event involving *atp*8 in the parasitic plant *Cynomorium* from Caryophyllales or Sapindales hosts (Cusimano and Renner 2019). Here, we identified a very short region (15 bp) in *atp*8 shared by Hydnoraceae, *Lactoris*, and nine other distantly related angiosperms, half of which are parasitic (*Cuscuta*, *Cynomorium*, *Tolypanthus*, and *Rhopalocnemis*) (fig. 3*A*). We, therefore, hypothesize that this short DNA fragment was frequently transferred (at least five times) between parasitic plants and their hosts because such HGT events have been identified in several distantly related lineages.

### Mutation Patterns of Perianth-Bearing Piperales Mitochondrial Genomes

**Fig. 4.**
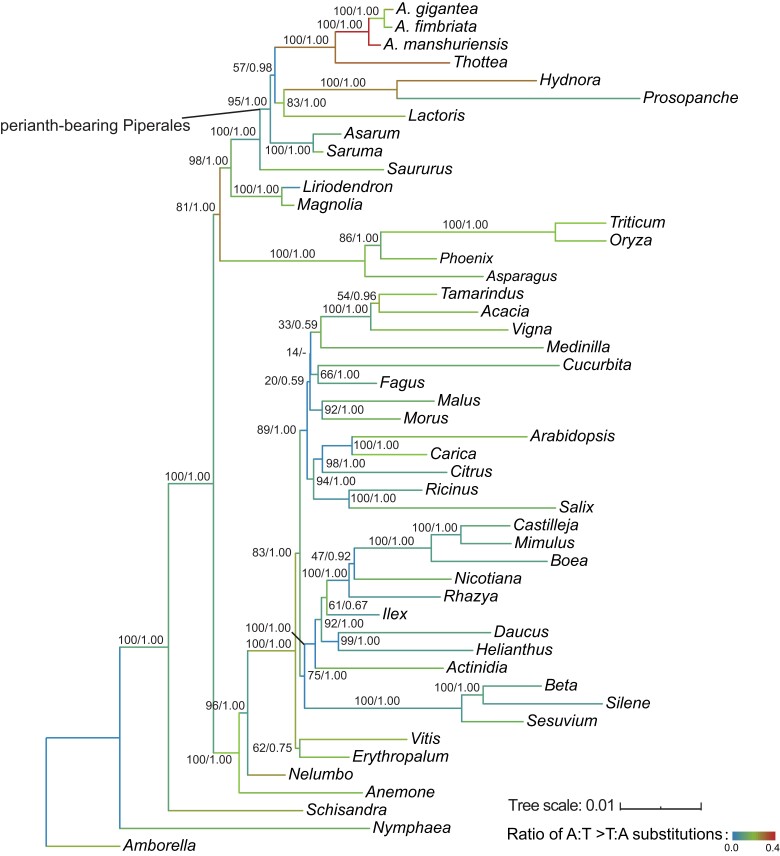
ML tree highlighting the mitochondrial mutation spectrum of 48 angiosperms. Enlarged view of the clade containing selected perianth-bearing Piperales studied in this analysis. Above each branch are bootstrap support values from ML and posterior probabilities from BI separated by a slash. RNA editing sites were excluded from the alignments. Branch colors indicate the proportion of A:T > T:A substitutions out of the total number of substitutions.

Mutation spectrum can be used to decipher the evolutionary history and mutation bias of genomic sequences but was rarely applied to plant mitochondrial sequences (Sloan and Wu 2014; Wu et al. 2020; Broz et al. 2021). To explore the mutation spectrum in angiosperms, we first analyzed phylogenetic relationships using a concatenated sequence matrix of the 24 core PCGs of 48 angiosperms (with the HGT regions in *atp*8 and *cox*1 excluded), and calculated the mutation spectrum accordingly (fig. 4 and supplementary table S8, Supplementary Material online). Overall, we found the frequency of C:G > T:A substitution was significantly higher than other substitution types [honestly significant difference (HSD) test, *P* value = 0] (supplementary fig. S6, Supplementary Material online and supplementary table S9, Supplementary Material online), confirming an AT-biased and transition-favored mutation pattern in plant mitochondria (Sloan and Wu 2014; Wu et al. 2020).

We then compared the mutation spectrum between perianth-bearing Piperales and other species. In total, 10,669 substitutions were identified with BASEML, 2,014 of which were distributed in the ancestral, internal, and external (tip) branches of the magnoliids (supplementary table S8, Supplementary Material online). We found biased enrichment of specific substitution types in different lineages of magnoliids (supplementary fig. S6, Supplementary Material online). For example, several of the branches that were most highly (top 10) enriched in C:G > A:T substitutions were the ancestor and external Asaraceae branches (Branches 78–80). Similar enrichment was also found for the A:T > C:G substitution type on the ancestral and external Hydnoraceae branches (Branches 74–76). We also found that the external branches of *Saururus* (Branch 81) and *Liriodendron* (Branch 83) showed enrichment of A:T > C:G and C:G > A:T substitutions, respectively. Notably, the ancestral *Thottea* and *Aristolochia* branches and the external *Thottea* branch (Branches 66, 67, and 72, respectively) showed the highest ratios of A:T > T:A substitutions of all branches (fig. 4 and supplementary fig. S6, Supplementary Material online). The A:T > T:A biased mutation has been also observed in AAs-treated animal cells (Hoang et al. 2013; Lai et al. 2022). It is interesting to further investigate whether the identified A:T > T:A mutation bias could also be associated with AAs.

Plant mitochondrial genomes often have highly dynamic structures and fast-evolving intergenic regions (Christensen 2013; Cole et al. 2018; Lin et al. 2022). To compare the rates of sequence evolution of the three nearly complete *Aristolochia* mitochondrial genomes, we classified all sequences as intergenic, exonic, intronic, or RNA gene regions, and calculated the rates for substitutions and indels (table 2). The HGT regions in *atp*8 and *cox*1 were excluded from the exonic dataset. Among the three mitochondrial genome pairs, exonic regions contained significantly lower substitution rates than intergenic regions (*t*-test, *P* value = 0.05). The significantly decreased *d*_N_ values in exons contributed to these discrepancies, whereas the *d*_S_ values have in significantly differed and slightly lower than the substitution rates in intergenic regions (*t*-test, *P* value = 0.15). Moreover, the mitochondrial PCGs exhibited significantly low evolutionary rates compared to the 17 nuclear oxidative phosphorylation (OXPHOS) genes (*t*-test, *P* value < 0.05), being consistent with previous studies (Drouin et al. 2008; Christensen 2013). Substitution rates in RNA genes and *cis*-spliced introns were consistently intermediate, between those of exonic and intergenic regions, except for the lower rate in RNA genes in the subgenus *Aristolochia.* In the pairwise comparisons, rates of sequence evolution were lowest in the subgenus *Aristolochia* for all four classes of sequence types. Additionally, our results showed extreme variations in evolutionary rates between the four sequence classes; notably, the intergenic region had a faster substitution and indel rate than synonymous sites in exonic regions. The differences between substitution rates in intergenic regions and synonymous sites could be due to overestimation of intergenic mutations (Fan et al. 2022). It could also suggest deleterious effects of some synonymous mutations similar to the recent studies in nuclear genomes (Shen et al. 2022).

**Table 2:** Substitution Rates in Nuclear Genes and Mitochondrial Genomes of Aristolochia Species

Pairwise comparison^a^	ts	tv	R (ts/tv)	Substitution rate (ts + tv)	*d*_N_	*d*_S_	ω (*d*_N_/*d*_S_)	indel^b^ rate
AriF versus AriG								
nu OXPHOS genes	2.68 × 10−2	1.59 × 10−2	1.69	4.27 × 10−2	1.49 × 10−2	1.13 × 10−1	0.13	0
mt exon regions	2.30 × 10−4	1.68 × 10−3	0.14	1.91 × 10−3	1.25 × 10−3	3.86 × 10−3	0.32	0
mt *cis*-spliced introns	3.41 × 10−4	2.00 × 10−3	0.17	2.34 × 10−3	—	—	—	1.24 × 10−4
mt RNA genes	2.94 × 10−4	1.03 × 10−3	0.29	1.32 × 10−3	—	—	—	0
mt intergenic regions	7.40 × 10−4	3.72 × 10−3	0.20	4.50 × 10−3	—	—	—	8.30 × 10−4
AriF versus AriM								
nu OXPHOS genes	6.88 × 10−2	4.55 × 10−2	1.51	1.24 × 10−1	3.63 × 10−2	3.18 × 10−1	0.11	7.10 × 10−5
mt exon regions	1.65 × 10−3	3.45 × 10−3	0.48	5.11 × 10−3	4.14 × 10−3	7.73 × 10−3	0.54	1.44 × 10−4
mt cis-spliced introns	2.13 × 10−3	8.75 × 10−3	0.24	1.09 × 10−2	—	—	—	1.09 × 10−3
mt RNA genes	3.58 × 10−3	8.21 × 10−3	0.44	1.12 × 10−2	—	—	—	7.33 × 10−4
mt intergenic regions	3.50 × 10−3	1.35 × 10−2	0.26	1.70 × 10−2	—	—	—	1.68 × 10−3
AriG versus AriM								
nu OXPHOS genes	7.47 × 10−3	4.88 × 10−2	1.53	1.14 × 10−1	3.86 × 10−2	3.46 × 10−1	0.11	7.10 × 10−5
mt exon regions	1.51 × 10−3	3.39 × 10−3	0.44	4.90 × 10−3	4.01 × 10−3	7.48 × 10−3	0.54	1.44 × 10−4
mt cis-spliced introns	2.22 × 10−3	8.36 × 10−3	0.27	1.06 × 10−2	—	—	—	1.09 × 10−3
mt RNA genes	3.43 × 10−3	8.06 × 10−3	0.43	1.15 × 10−2	—	—	—	7.33 × 10−4
mt intergenic regions	3.56 × 10−3	1.36 × 10−2	0.26	1.71 × 10−2	—	—	—	2.14 × 10−3

a AriF, *A. fimbriata*; AriG, *A. gigantea*; AriM, *A. manshuriensis*.

b Insertion/deletion mutations (indels) ≤20 bp.

## Conclusions

**Fig. 5.**
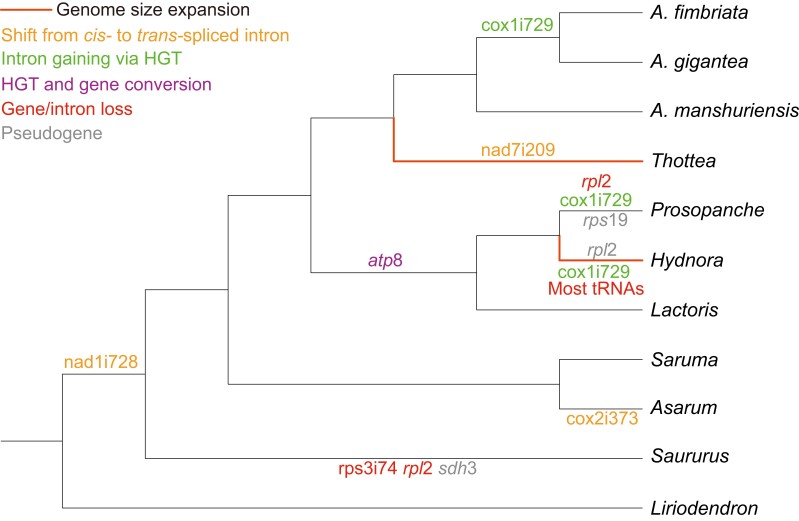
Summary of mitochondrial genome features in magnoliids.

In summary, we here assembled and characterized the features of mitochondrial genomes in perianth-bearing Piperales. Comparative analyses of these mitochondrial genomes show genome size heterogeneity, tRNA gene variation, shifts from *cis*- to *trans*-splicing of Group II introns, HGT in *cox*1 and *atp*8 genes, as well as enrichment of certain substitution types in Piperales (fig. 5). In general, we found most of these features seem to be diversified within magnoliids. Therefore, this study not only helps with a better understanding of mitochondrial genomes in magnoliids but also provides important resources for deciphering the evolution of angiosperm mitochondrial genomes more broadly.

## Materials and Methods

### Sampling, Plant Materials, and Sequencing

Representatives of all perianth-bearing Piperales genera were sampled. Additional mitochondrial genomes of various other angiosperms were included for comparison (supplementary table S10, Supplementary Material online). Fresh plant material was collected from Aristolochiaceae and Asaraceae species grown in greenhouses at the Institute of Botany, Chinese Academy of Sciences, and complemented with material from the field. Illumina and Nanopore read of the *A. fimbriata* genome from a previous study by our lab (Qin et al. 2021) were used; these data are available under the GenBank BioProject number PRJNA656149. For Hydnoraceae and Lactoridaceae, Illumina data generated in previous studies were used (Naumann et al. 2016; Jost et al. 2020, 2021). For the five other species (*A. gigantea*, *A. manshuriensis*, *Thottea*, *Asarum*, and *Saruma*), total DNA was extracted with a Hipure Plant DNA Mini Kit (Magen, Shenzhen, China). Shotgun genomic libraries were constructed with a 350 bp insert size, then sequenced on the Illumina NovaSeq platform. This yielded 11.3 Gb of 150-bp paired-end reads for newly sequenced Aristolochiaceae and Asaraceae. For *Saururus chinensis*, Illumina reads were downloaded from GenBank (accession number ERR3412413) (Xue et al. 2022). Trimmomatic v0.39 (Bolger et al. 2014) was used with default parameters to filter all Illumina reads before downstream analyses.

### Mitochondrial Genome Assembly and Annotation

We first assembled a draft mitochondrial genome of *A. fimbriata* with GetOrganelle v1.7.1 (Jin et al. 2020) using the “embplant_mt” option and a custom angiosperm mitochondrial database (supplementary table S10, Supplementary Material online) as reference. This draft was made of 37 contigs and totaled 338,601 bp in length. The contigs were used as seeds to extract mitochondrial Nanopore reads with BLASR v5.3.3 (Chaisson and Tesler 2012) with the parameters minAlnLength and minPctSimilarity set to 2000 and 75, respectively (parameters “P1”). We then used Canu v1.8 (Koren et al. 2017) to self-correct and trim putative mitochondrial Nanopore reads. Corrected reads were used as seeds to perform a second round of extraction and correction. In this second round, minAlnLength and minPctSimilarity were set to 5000 and 90, respectively (parameters “P2”). This yielded ∼170 Mb (10,958) of high-confidence mitochondrial Nanopore reads, which were used as input along with the Illumina reads in Unicycler v0.4.9 (Wick et al. 2017) using default parameters. The complete mitochondrial genome of *A. fimbriata* was then assembled into three circular contigs. Using the *A. fimbriata* mitochondrial genome as the reference, mitochondrial genomes were assembled for *A. gigantea*, *A. manshuriensis*, *Prosopanche*, *Asarum*, *Saruma*, *Saururus*, and *Thottea* using GetOrganelle as previously described (Yu et al. 2021). Additionally, we mapped the Illumina DNA-seq reads to the mitochondrial genomes of three *Aristolochia* species using Bowtie2 v2.4.0 (Langmead and Salzberg 2012), using the parameters “--end-to-end --no-discordant --no-mixed.” The depth of coverage throughout each mitochondrial genome was calculated with Samtools v1.9 (Li et al. 2009).

For *Hydnora*, potential mitochondrial PacBio reads were first identified with BLASR using the same angiosperm mitochondrial references and parameters “P1”. Identified PacBio reads were corrected, trimmed, and assembled with Canu using default parameters. We then used BLASTN with “-evalue 1e-5” and the custom mitochondrial dataset to select contigs containing at least one mitochondrial gene. These contigs were used as the seed to identify putative mitochondrial PacBio reads from the corrected PacBio reads with BLASR for a second round of assembly, using parameters “P2”. After seven rounds of assembly with parameters “P2”, no additional reads were identified, and we obtained 16 linear contigs totaling 1,916,195 bp in length.

To annotate PCGs and rRNAs in our newly assembled mitochondrial genomes, we employed a BLASTN search with angiosperm mitochondrial genes (supplementary table S10, Supplementary Material online) as a query, using the sensitive parameters described by Skippington et al. (2017). *Trans*-spliced introns were identified as described by Guo et al. (2020). tRNA annotation was conducted with tRNAscan-SE v2.0 (Chan et al. 2021) in “organelle” mode and a sensitive BLASTN search using a custom angiosperm tRNA database.

### Plastid Genome Assembly and Analysis of Mitochondrial Plastid-Derived Sequences (MTPTs)

Illumina DNA-seq reads corresponding to *A. gigantea* and *Thottea* plastids were assembled with the “embplant_pt” option in GetOrganelle. Plastid genomic sequences of *A. fimbriata*, *A. manshuriensis*, *Hydnora*, *Prosopanche*, *Asarum*, *Saruma*, and *Saururus* were downloaded from GenBank (accession numbers CM034085, NC_046766, NC_029358, MT075717, MW034667, NC_039933, and NC_050853, respectively). For each species, we performed BLASTN searches with “-evalue 1e-5” and “-perc_identity 80” to search for any MTPTs longer than 100 bp in the assembled mitochondrial contigs, using the Plastid genome of each species as the reference. For the parasitic *Prosopanche* and *Hydnora* species, the Plastid genomes of their hosts, *Prosopis cineraria* (accession number NC_049133) and *Euphorbia hirta* (accession number NC_058203), were added to the references, as was *A. fimbriata*. We further used PGA (Qu et al. 2019) to annotate genes derived from the plastid genome by comparing the mitochondrial genomes with reference plastid genomes as described above with the parameter “-p 80”. The PGA output file (warning.log) was analyzed to detect putative pseudogenes.

### Identification of Interspersed Repeats and Evaluation of Repeat-Mediated Recombination

We used the ROUSFinder2.py script written by Wynn and Christensen (2019) to search the mitochondrial genomes for interspersed repeats (≥50 bp). Wilcoxon rank sum test was performed to compare the repeat density between mitochondrial genomes of *Aristolochia* and other angiosperms. We then assessed the recombination activity for each repeat pair. Using the 300-bp upstream and downstream flanking sequences, four references, including the two genomic sequences and two corresponding alternative conformations, were constructed for each pair of repeats. For repeats <350 bp (the insert size of our Illumina library), Bowtie2 was used to map all Illumina DNA-seq reads using the same parameters. For repeats >350 bp in *A. fimbriata*, the length of the flanking sequences was changed to 1000 bp and Nanopore long reads were mapped to the references using minimap2 (Li 2018) where possible. Finally, the number and percentage of reads supporting either reference or alternative conformations of repeat pairs were counted.

### Synteny Analysis and Calculation of Substitution Rates

BLASTN was used to identify synteny between the three *Aristolochia* species with the following parameters: -evalue 1e-5 -perc_identity 80. In the first round, only matches longer than 250 bp were retained. Syntenic regions were then further divided into four groups: exons, introns, RNA genes, and intergenic regions. For exons, amino acid sequences were aligned using MAFFT v7.427 (Katoh and Standley 2013) with the L-INS-I algorithm. Amino acid alignments were then converted to nucleotide alignments using PAL2NAL v14 (Suyama et al. 2006). Nucleotide sequences of other regions were aligned with MUSCLE v3.8.31 (Edgar 2004). Rates of transition (ts), transversion (tv), and insertions/deletions (indels) (<20 bp) were determined for all regions, and the nonsynonymous (*d*_N_) and synonymous (*d*_S_) substitution rates for exons were calculated using “Distance Computation” module in MEGA X (Kumar et al. 2018) using the Tamura-Nei model. In addition, a total of 17 nuclear genes encoding OXPHOS that often target the mitochondria were identified in *Aristolochia*, and sequences of each studied OXPHOS gene in Aristolochia were deposited to the figshare (doi: 10.6084/m9.figshare.22152245.v1) online database. We then calculated pairwise substitution and indel mutation rates between the three species. *T*-tests were performed to compare the substitution rates among regions when needed. In *A. fimbriata*-*A. gigantea* species pair, we investigated the sequence content of each *A. fimbriata*-specific region of > 250 bp in detail. Besides checking the MTPT and repetitive content, we blasted these regions against mitochondrial genomes of other species which were used for annotation in this study (supplementary table S10, Supplementary Material online). Any hit with a >100 bp size and a >80 identity value was recorded.

### Phylogenetic Analyses

Mitochondrial genomes of additional 38 phylogenetically diverse angiosperms were downloaded from GenBank (supplementary table S10, Supplementary Material online). Sequences were aligned as described above. RNA editing sites were adopted from Edera et al. (2018) (supplementary table S10, Supplementary Material online), and these RNA editing sites were excluded from the alignments. Phylogenetic trees were constructed in RAxML-ng v0.9 (Kozlov et al. 2019) using the maximum likelihood (ML) method, the GTR + G model, and 1,000 bootstrap replicates. The best-fitting nucleotide substitution model was estimated before using ModelFinder (Kalyaanamoorthy et al. 2017), implemented in IQ-TREE.

Potential chimeric genes were identified in GENECONV v1.81 (Sawyer 1989) with the parameters “-Gscale = 3 -Minlength = 1 –pairwise”. For the *atp*8 gene, we checked all available homologs in GenBank (https://www.ncbi.nlm.nih.gov/nuccore). Additional *atp*8 genes from other angiosperms that showed signs of gene conversion were also included in the alignment (supplementary table S10, Supplementary Material online). We then downloaded the sequences of *cox*1 and the associated intron (cox1i729) from 111 diverse angiosperms (supplementary table S11, Supplementary Material online). Intron sequences were aligned with MUSCLE, followed by manual adjustment as necessary. Phylogenetic gene trees were constructed as detailed above. After removing the regions with gene conversion signals, we constructed a phylogenetic tree from the concatenated alignment of 24 mitochondrial core PCGs (Mower et al. 2012) from 48 angiosperms using the same methods. The topology was assessed using the Bayesian inference (BI) method with a GTR + G model in MrBayes v. 3.2.7 (Ronquist et al. 2012). Based on the topology of this tree, we further used the BASEML module in PAML v4.9 (Yang 2007) with default parameters to calculate the mutation spectrum in each branch. A boxplot was drawn to show variations of angiosperm mutation spectrum, including branches with at least 20 substitutions. Tukey's honestly significant difference (HSD) test was used to test the degree of difference among substitution types.

## Supplementary Material

evad041_Supplementary_Data

## Data Availability

All the raw sequencing reads generated in this study were deposited at the National Center for Biotechnology Information (NCBI) under the BioProject accession no. PRJNA888954. All the assembled and annotated mitochondrial genomes were submitted to GenBank under accession nos. OP649449–OP649552. The coding sequences of newly assembled mitochondrial genomes in this study have been deposited to the figshare online database (doi: 10.6084/m9.figshare.22152245.v1).
