# Supplementary material for: De novo Assembly and Comparative Analyses of Mitochondrial Genomes in Piperales
Source: Genome Biol Evol. 2023 Mar 10;15(3):evad041. doi: 10.1093/gbe/evad041 (PMC10036691; doi:10.1093/gbe/evad041)
Supplement: evad041_Supplementary_Data [file evad041_supplementary_data.zip › Supplementary figures 0116.pdf]

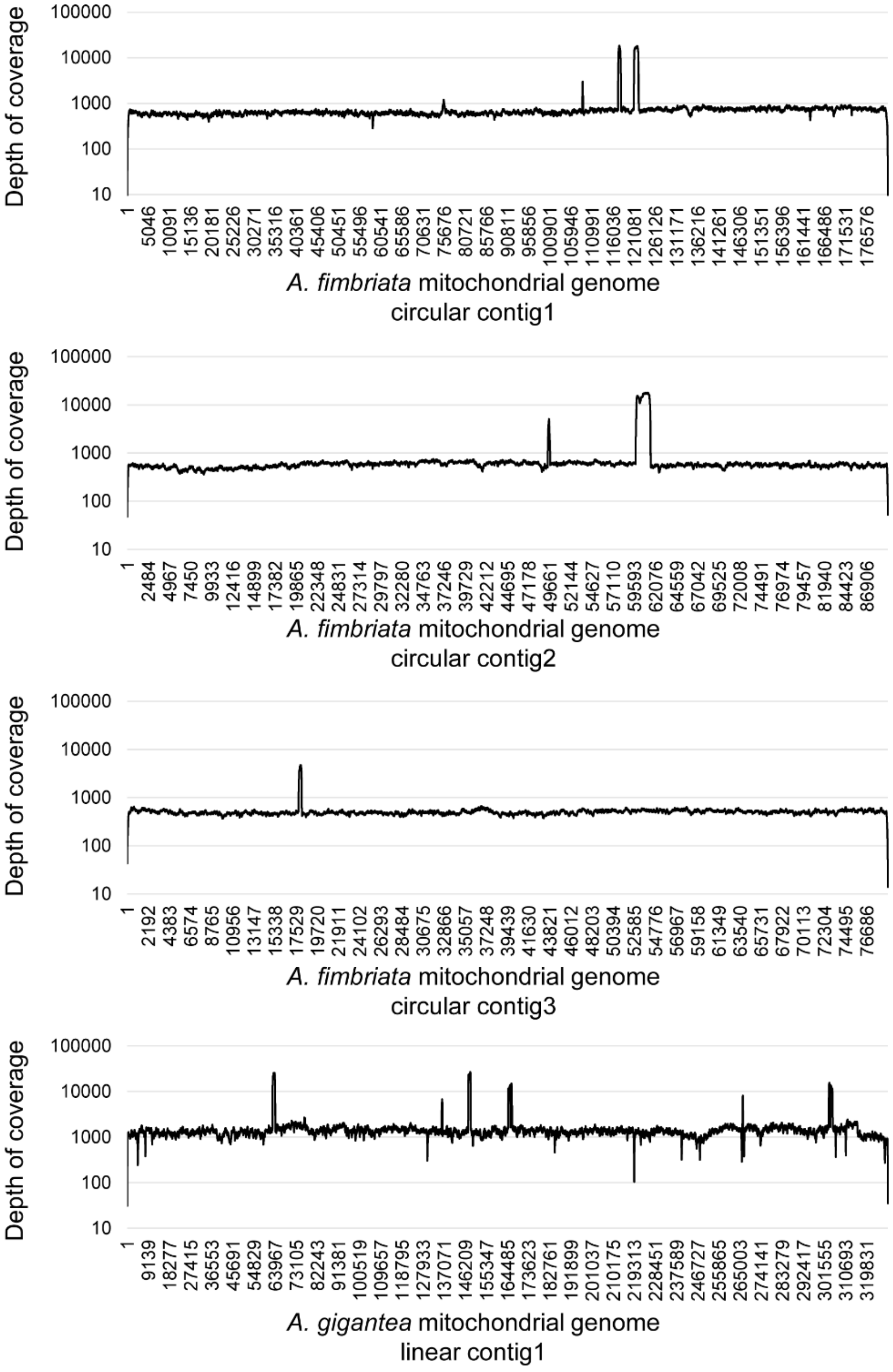

Fig. S1 Depth of Illumina DNA-seq coverage of assembled contigs for the three *Aristolochia* mitochondrial genomes.

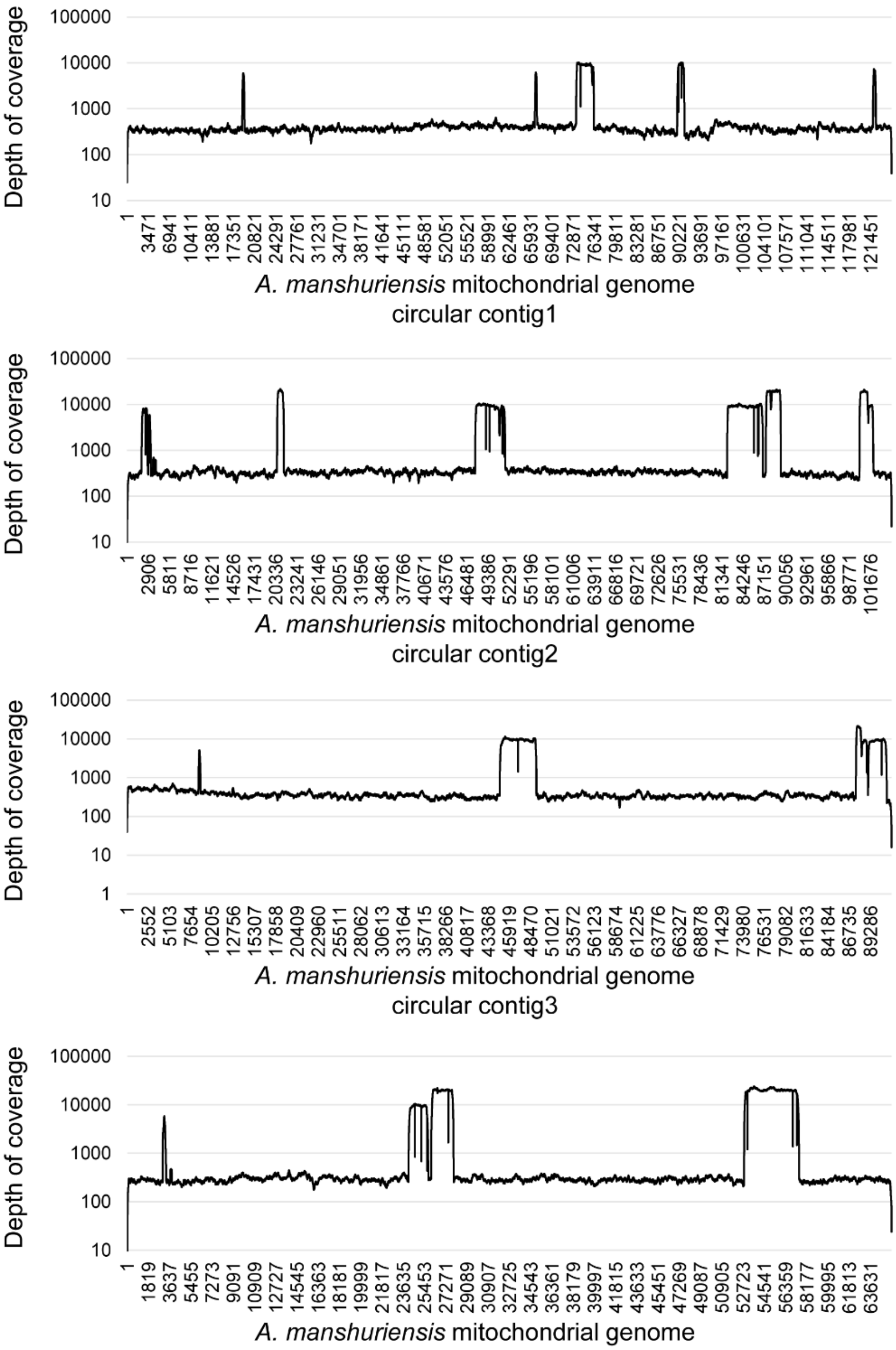

Fig. S1 Depth of Illumina DNA-seq coverage of assembled contigs for the three *Aristolochia* mitochondrial genomes.

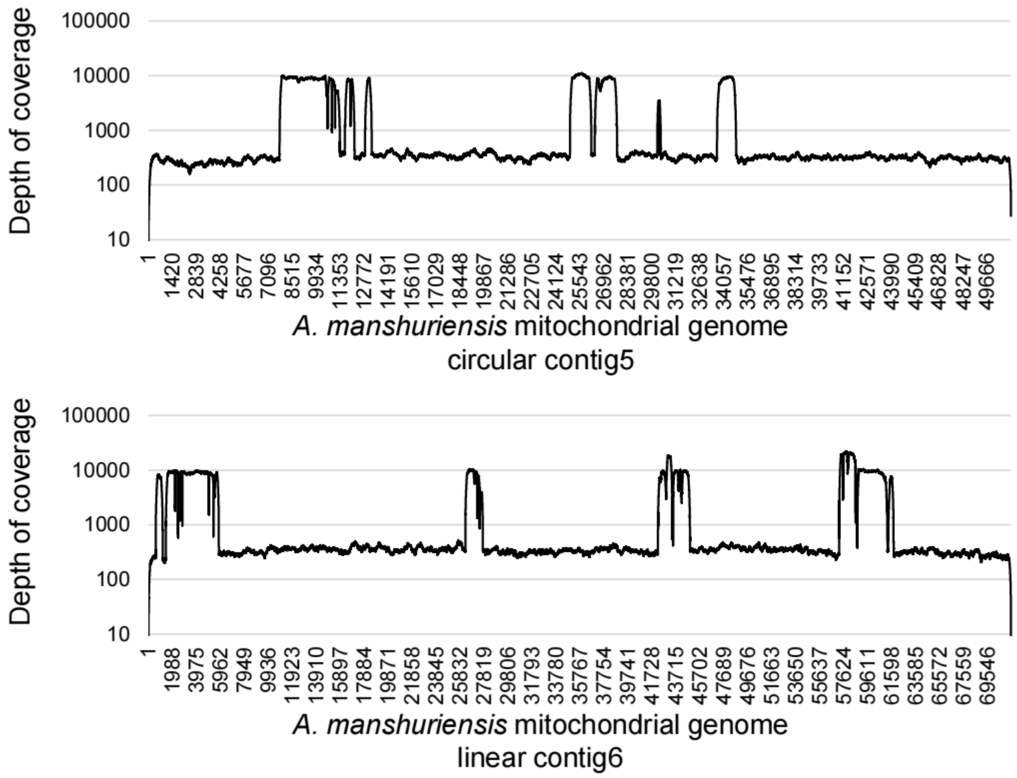

Fig. S1 Depth of Illumina DNA-seq coverage of assembled contigs for the three *Aristolochia* mitochondrial genomes.

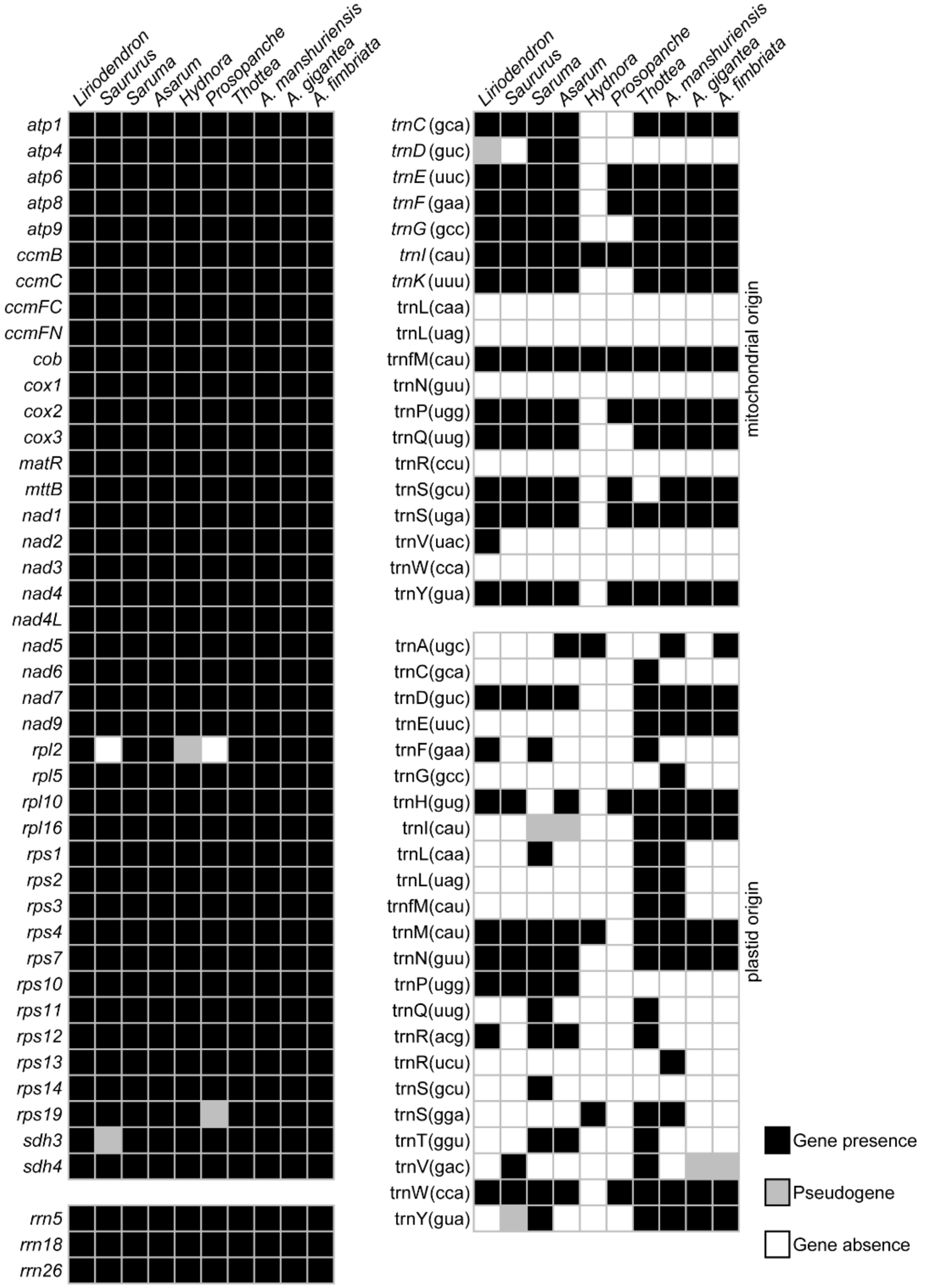

Fig. S2 Gene content in ten magnoliid mitochondrial genomes.

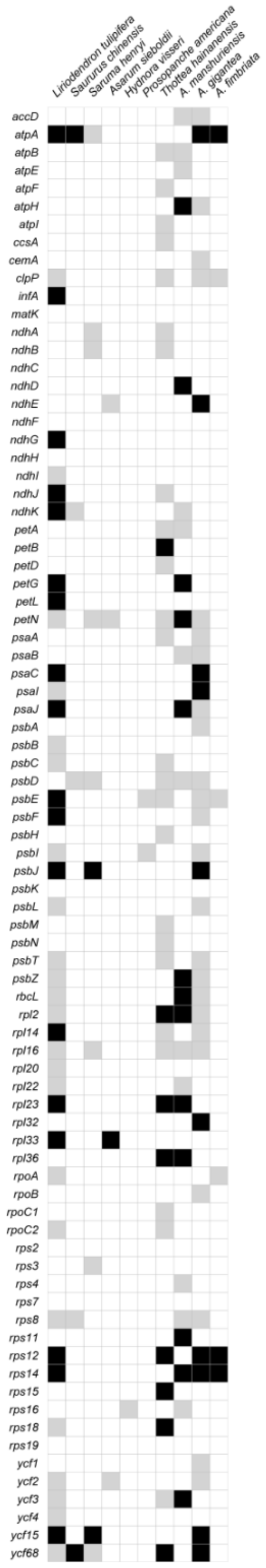

Fig. S3 Plastid-derived protein-coding gene content in ten magnoliid mitochondrial genomes.

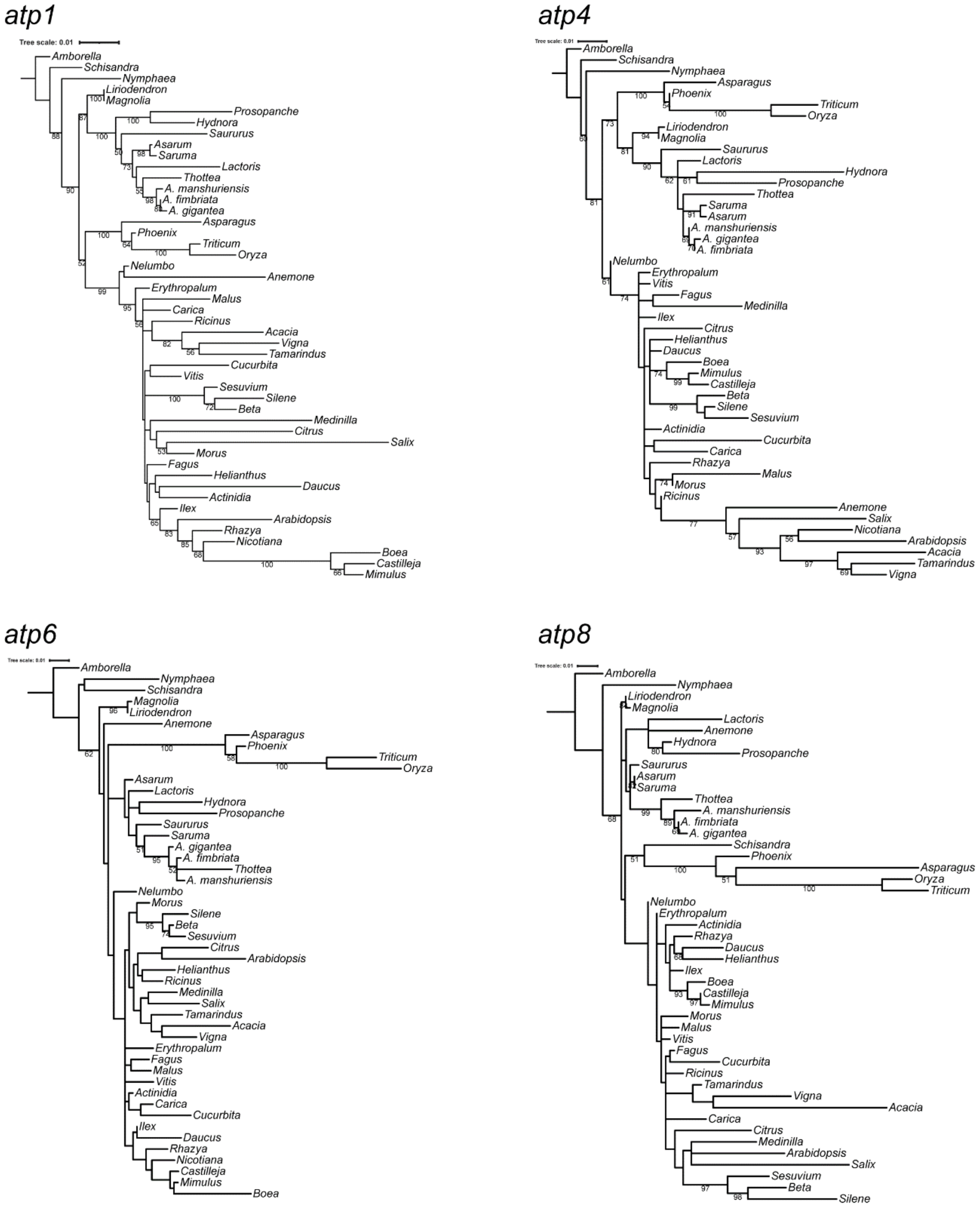

Fig. S4 Maximum likelihood phylogenetic trees showing relationships between 48 angiosperms. Bootstrap support values > 50 are shown above each branch. RNA editing sites were excluded from the alignments.

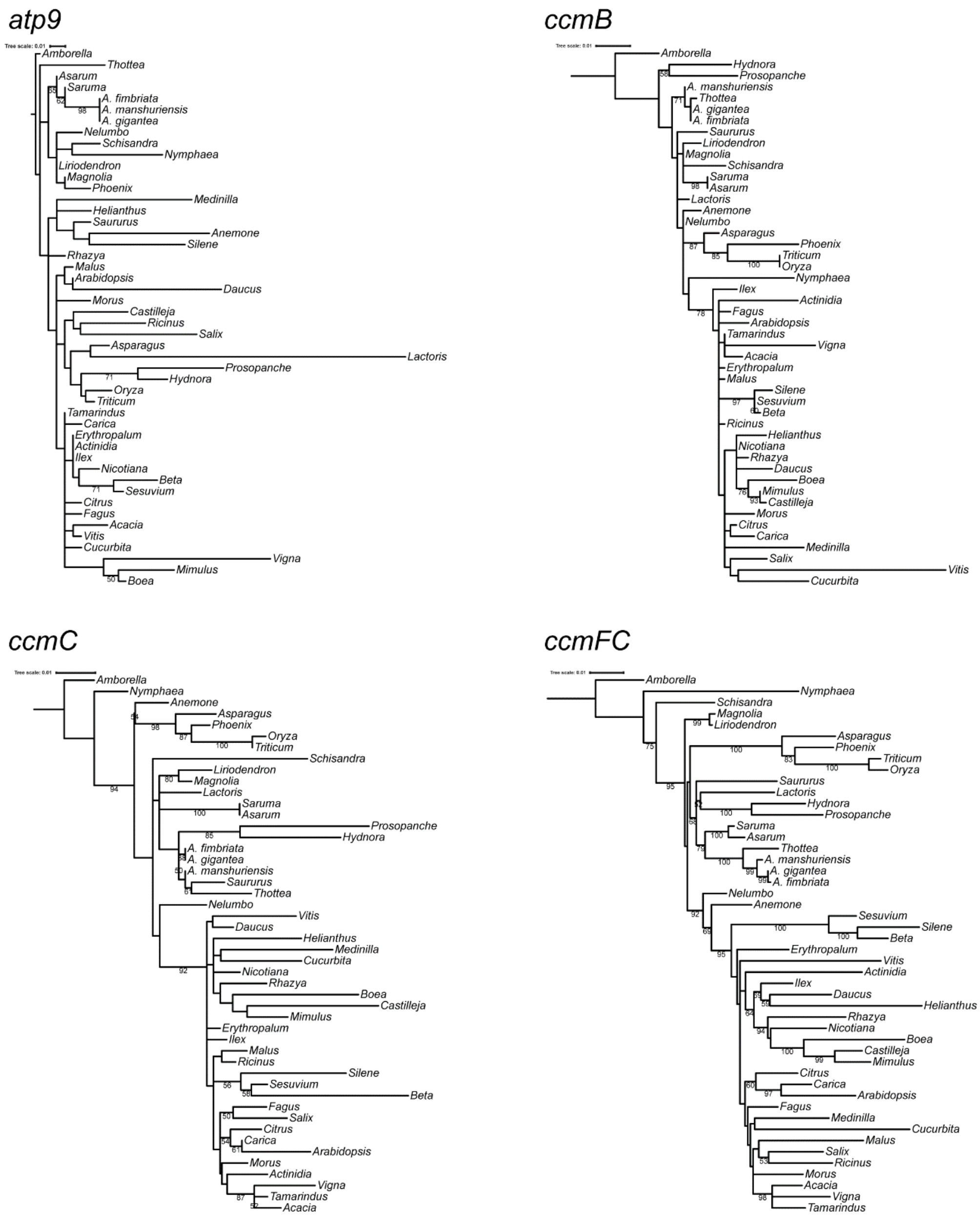

Fig. S4 Maximum likelihood phylogenetic trees showing relationships between 48 angiosperms. Bootstrap support values > 50 are shown above each branch. RNA editing sites were excluded from the alignments.

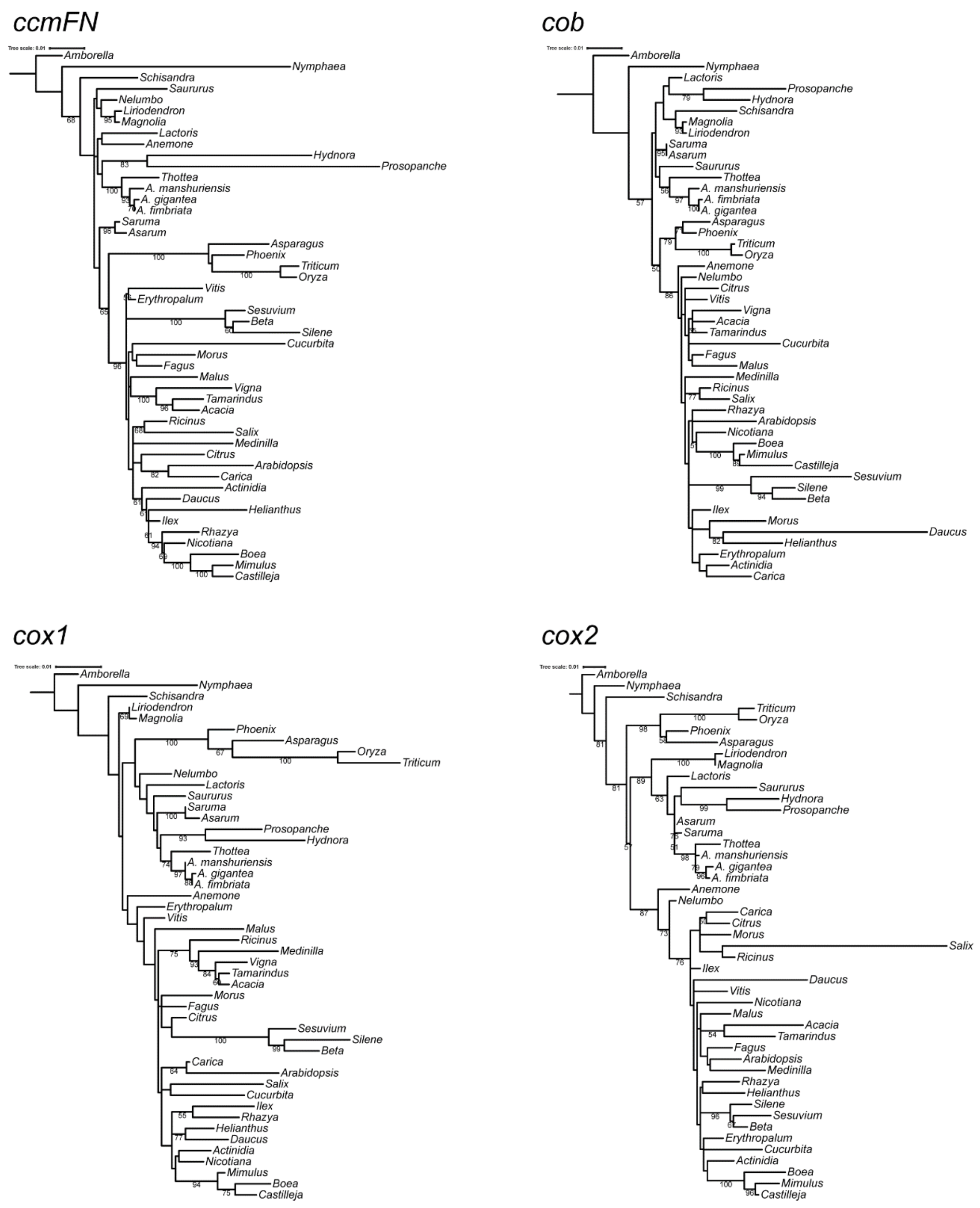

Fig. S4 Maximum likelihood phylogenetic trees showing relationships between 48 angiosperms. Bootstrap support values > 50 are shown above each branch. RNA editing sites were excluded from the alignments.

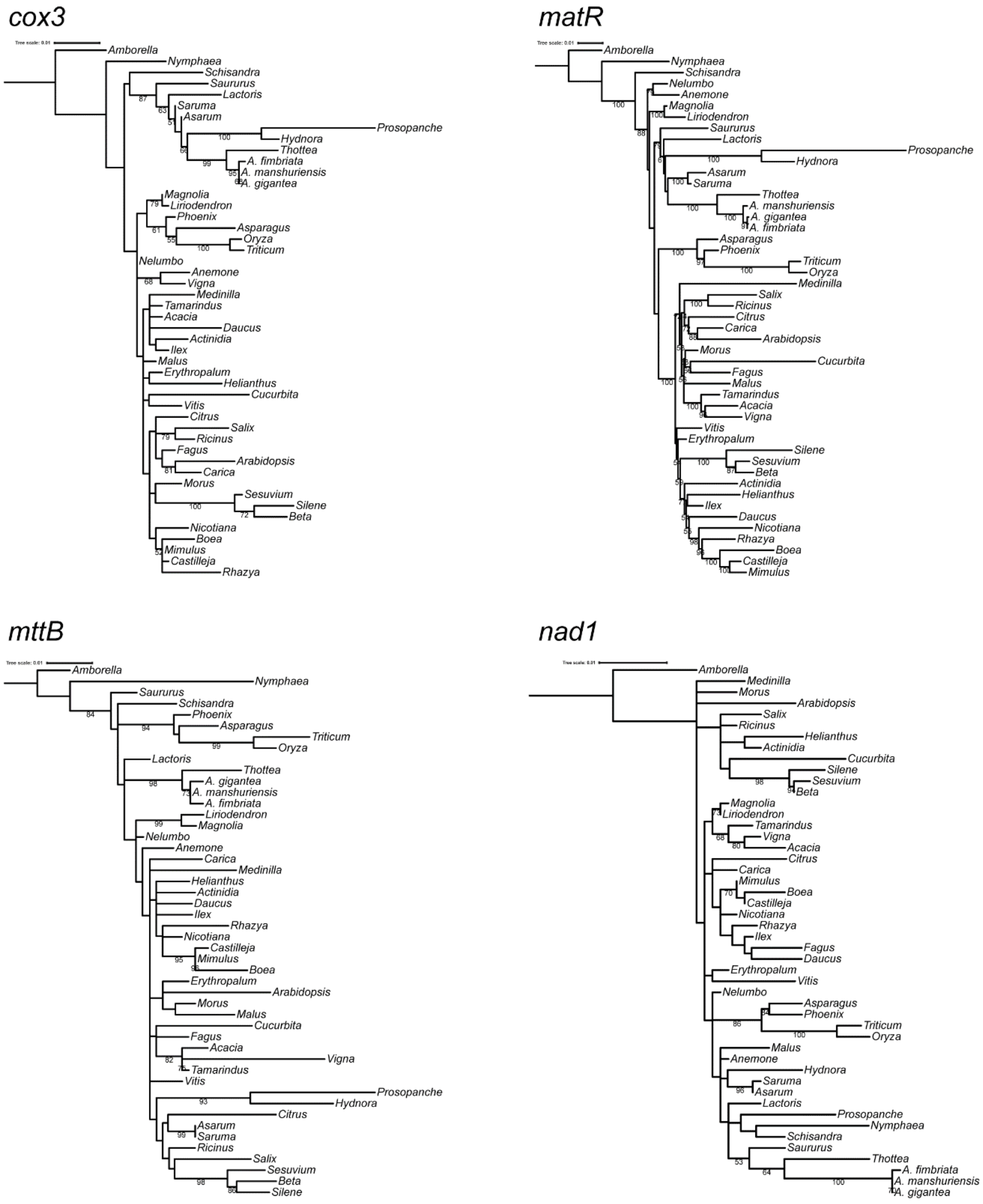

Fig. S4 Maximum likelihood phylogenetic trees showing relationships between 48 angiosperms. Bootstrap support values > 50 are shown above each branch. RNA editing sites were excluded from the alignments.

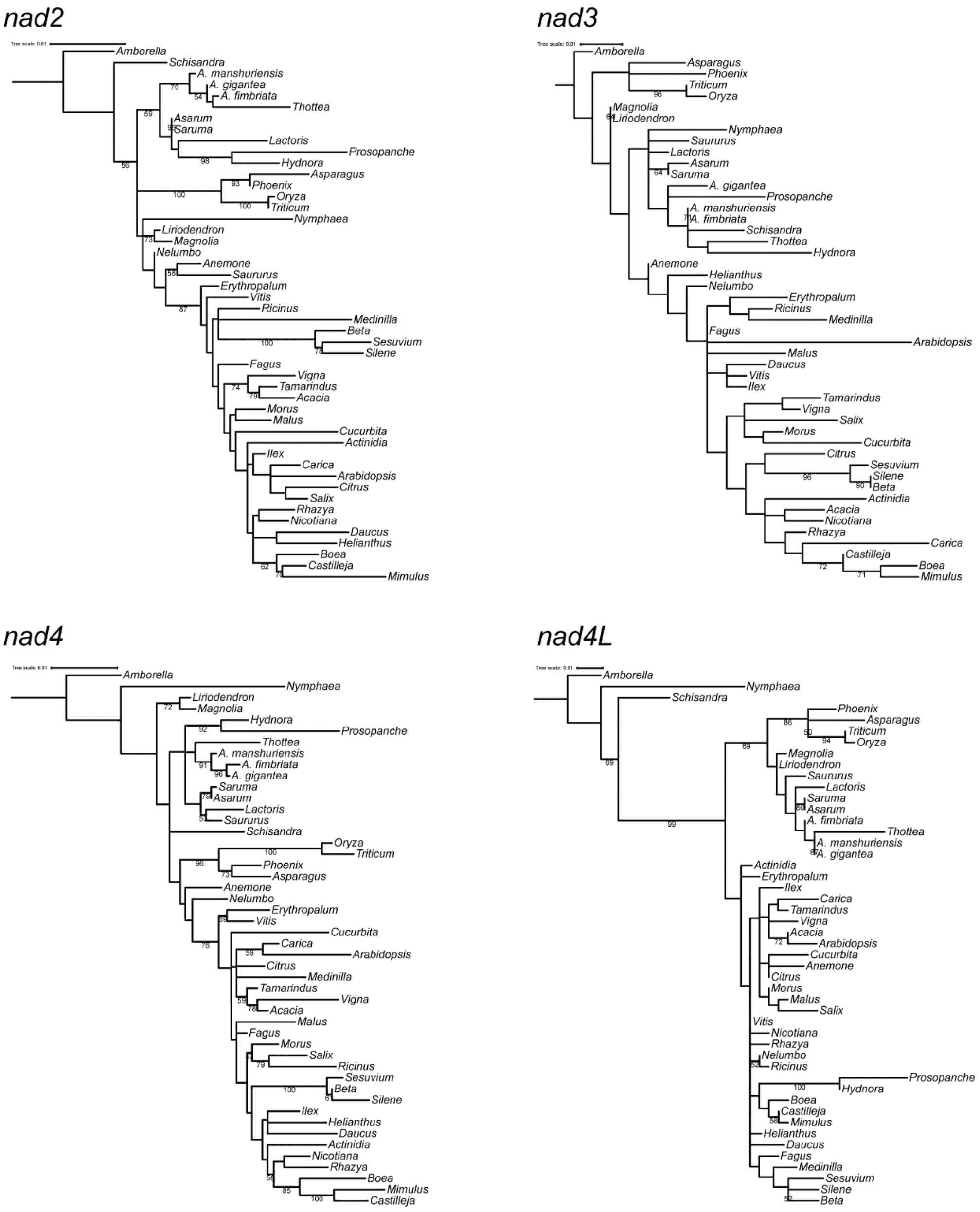

Fig. S4 Maximum likelihood phylogenetic trees showing relationships between 48 angiosperms. Bootstrap support values > 50 are shown above each branch. RNA editing sites were excluded from the alignments.

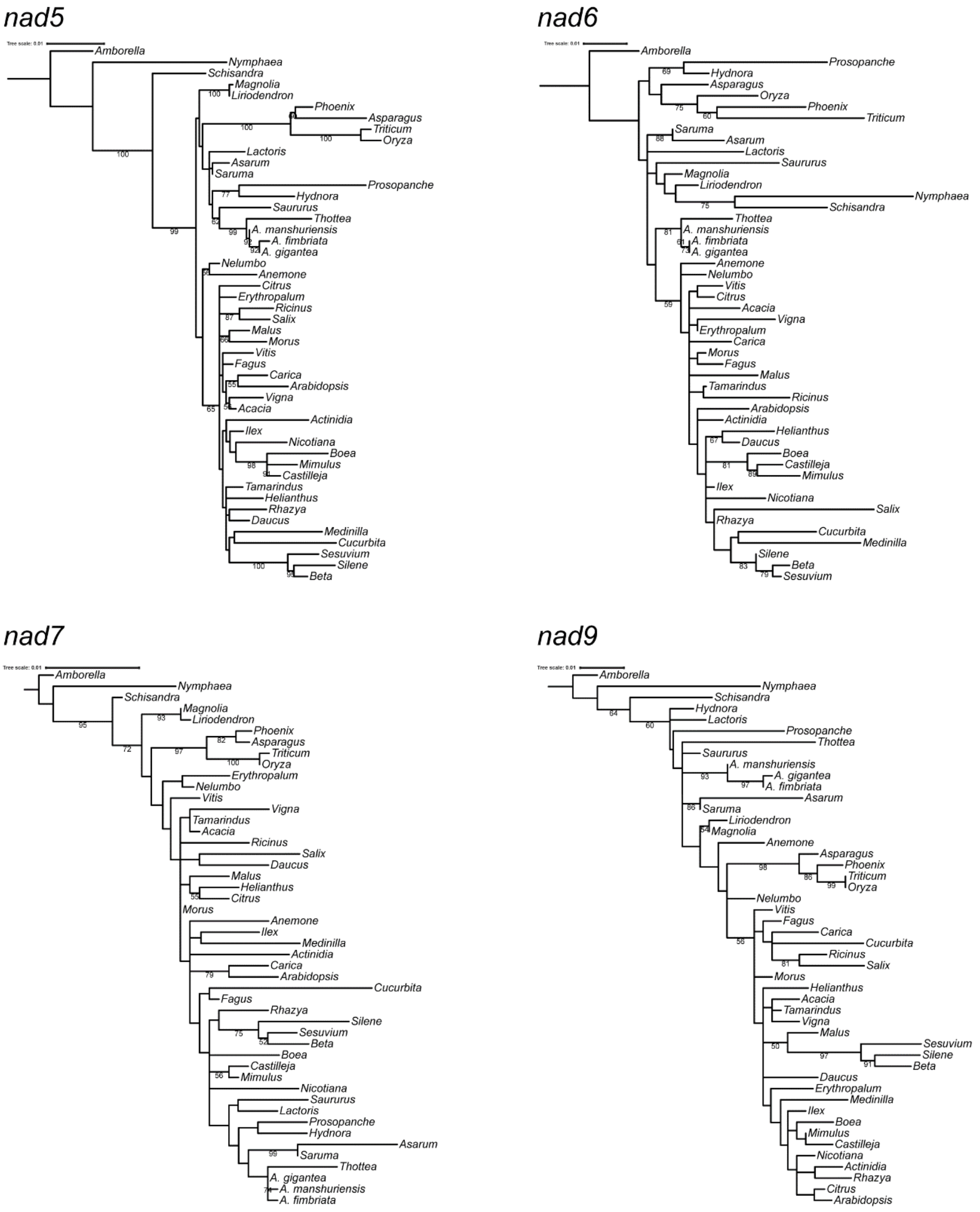

Fig. S4 Maximum likelihood phylogenetic trees showing relationships between 48 angiosperms. Bootstrap support values > 50 are shown above each branch. RNA editing sites were excluded from the alignments.

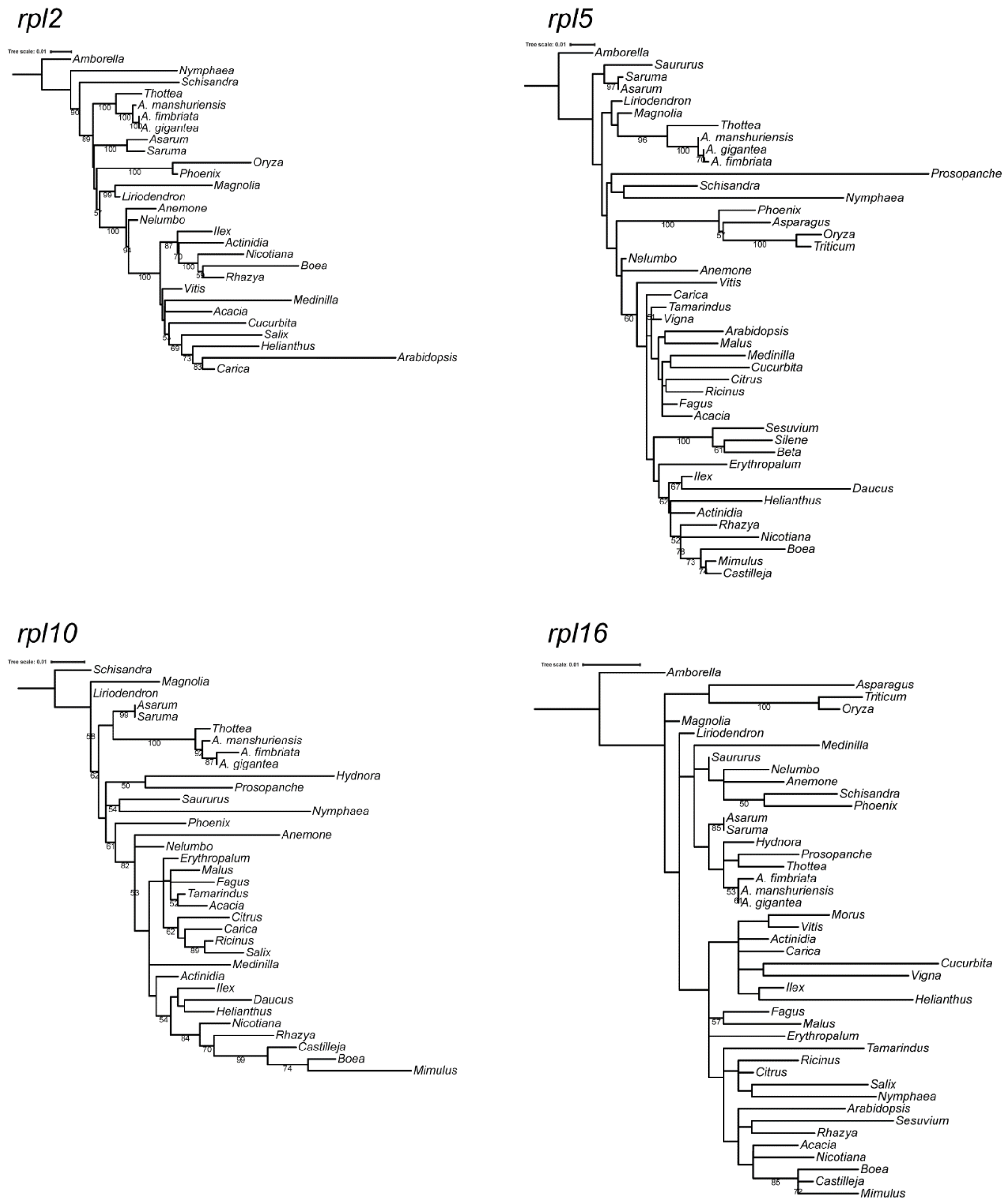

Fig. S4 Maximum likelihood phylogenetic trees showing relationships between 48 angiosperms. Bootstrap support values > 50 are shown above each branch. RNA editing sites were excluded from the alignments.

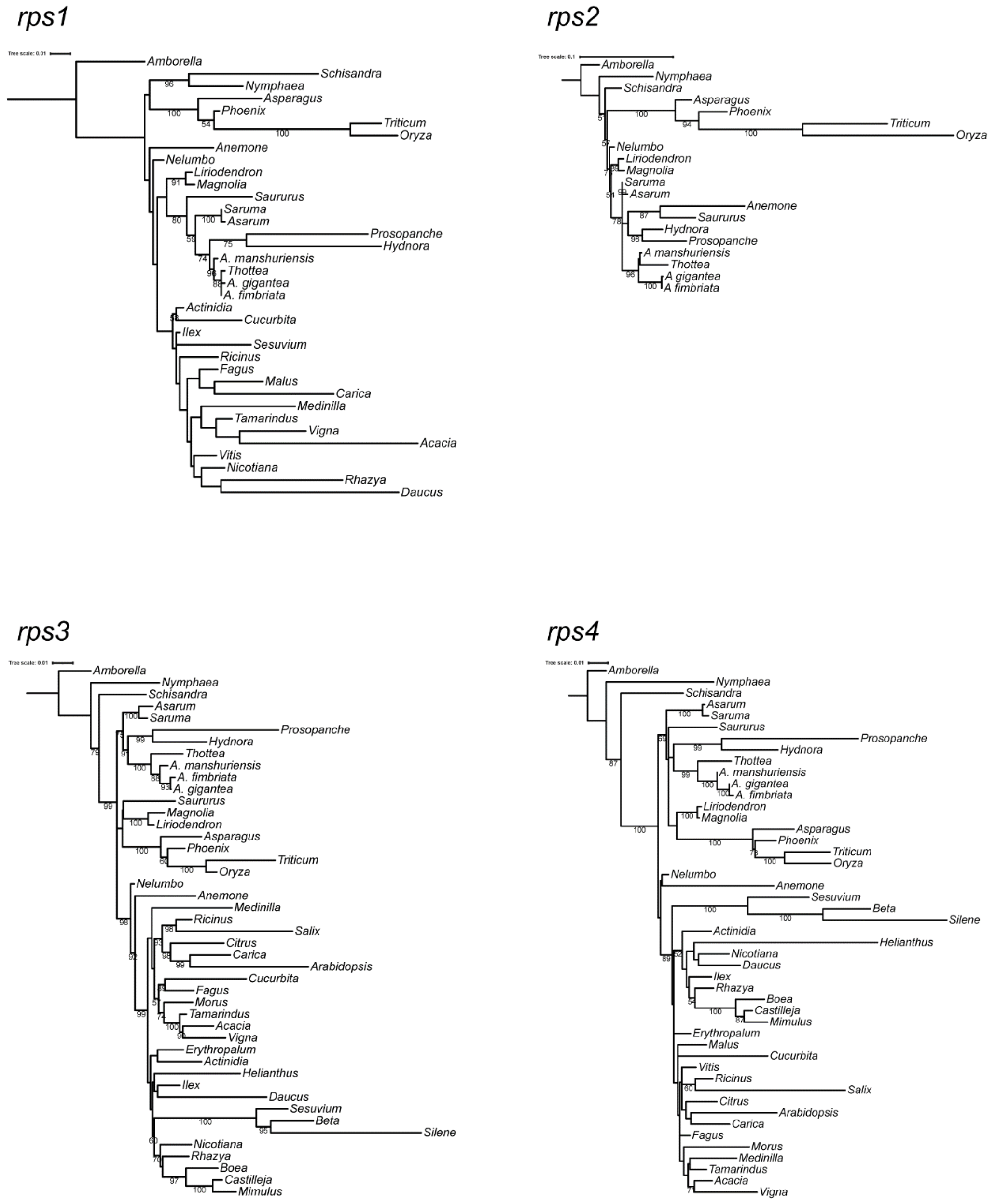

Fig. S4 Maximum likelihood phylogenetic trees showing relationships between 48 angiosperms. Bootstrap support values > 50 are shown above each branch. RNA editing sites were excluded from the alignments.

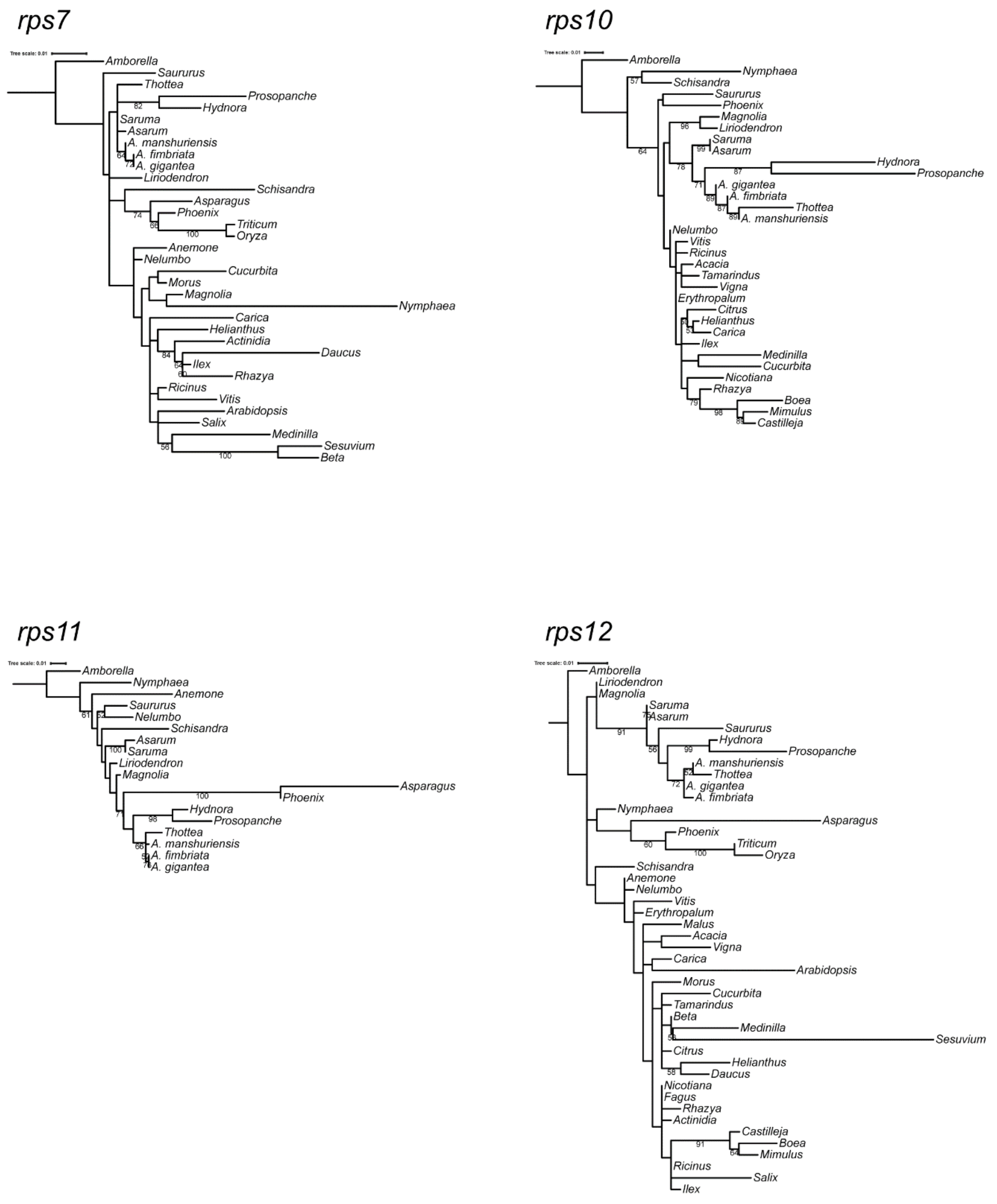

Fig. S4 Maximum likelihood phylogenetic trees showing relationships between 48 angiosperms. Bootstrap support values > 50 are shown above each branch. RNA editing sites were excluded from the alignments.

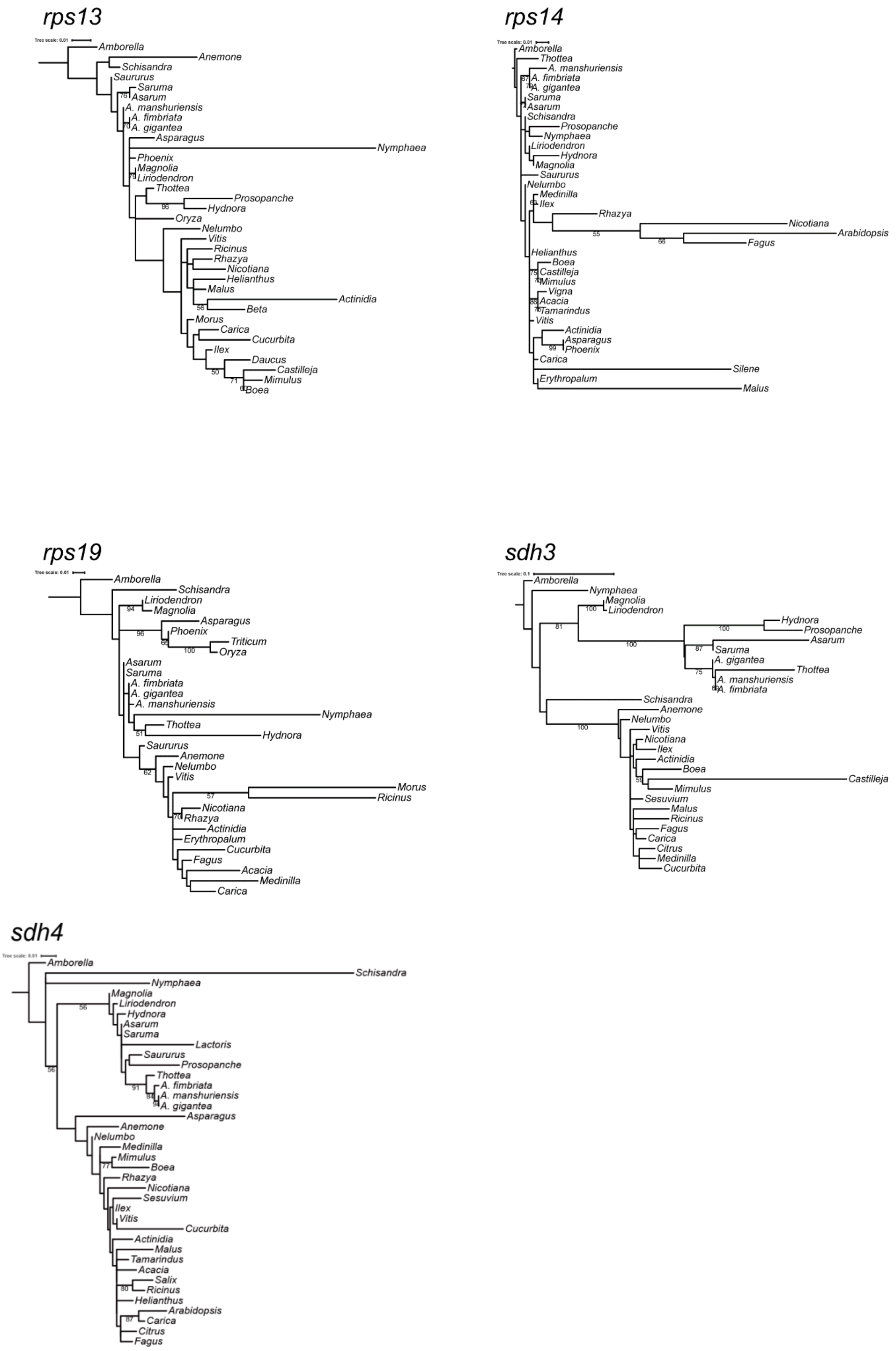

Fig. S4 Maximum likelihood phylogenetic trees showing relationships between 48 angiosperms. Bootstrap support values > 50 are shown above each branch. RNA editing sites were excluded from the alignments.

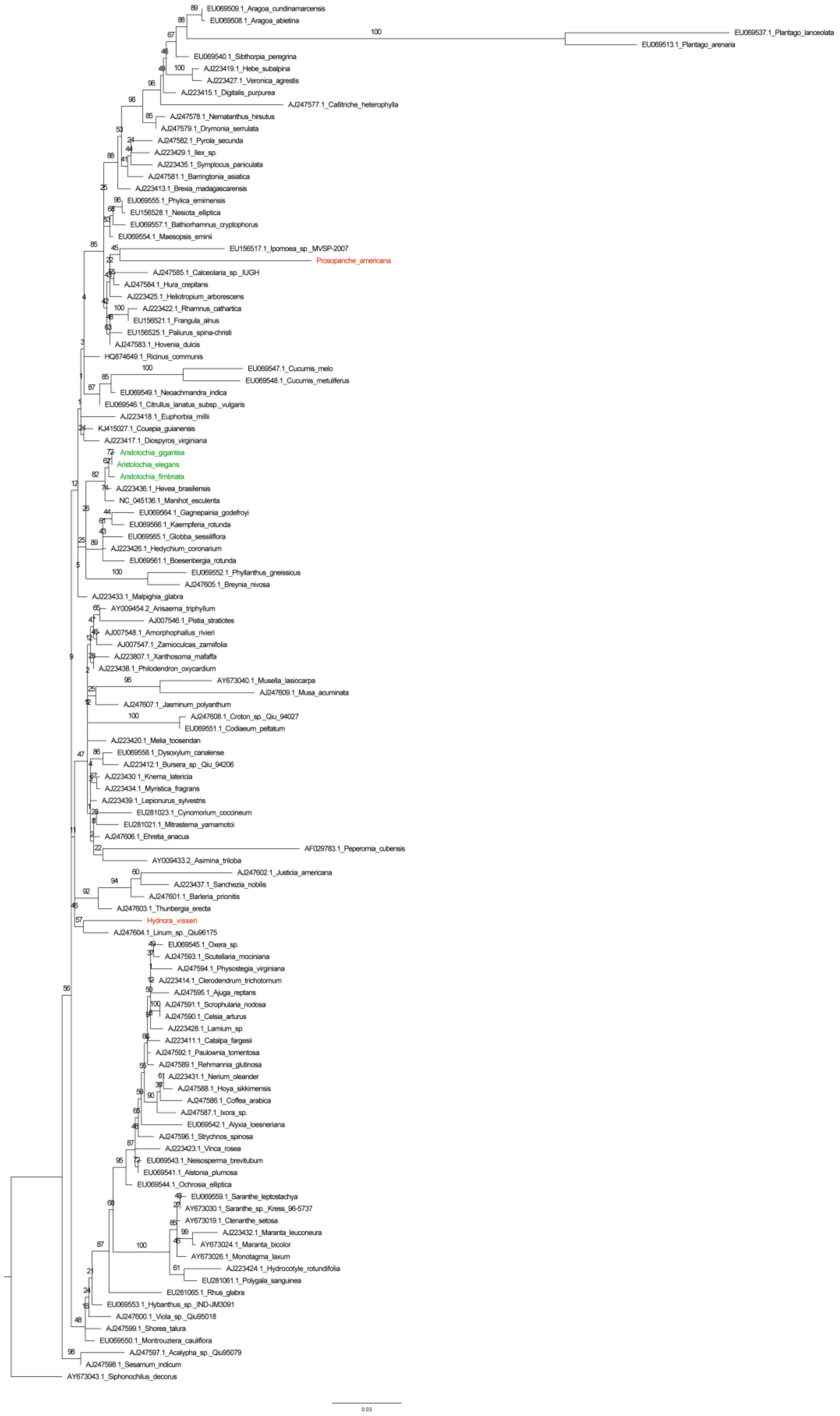

Fig. S5 Maximum likelihood phylogenetic trees showing relationships based on the *cox*1 intron (cox1i729) in many angiosperms. Bootstrap support values are shown above each branch.

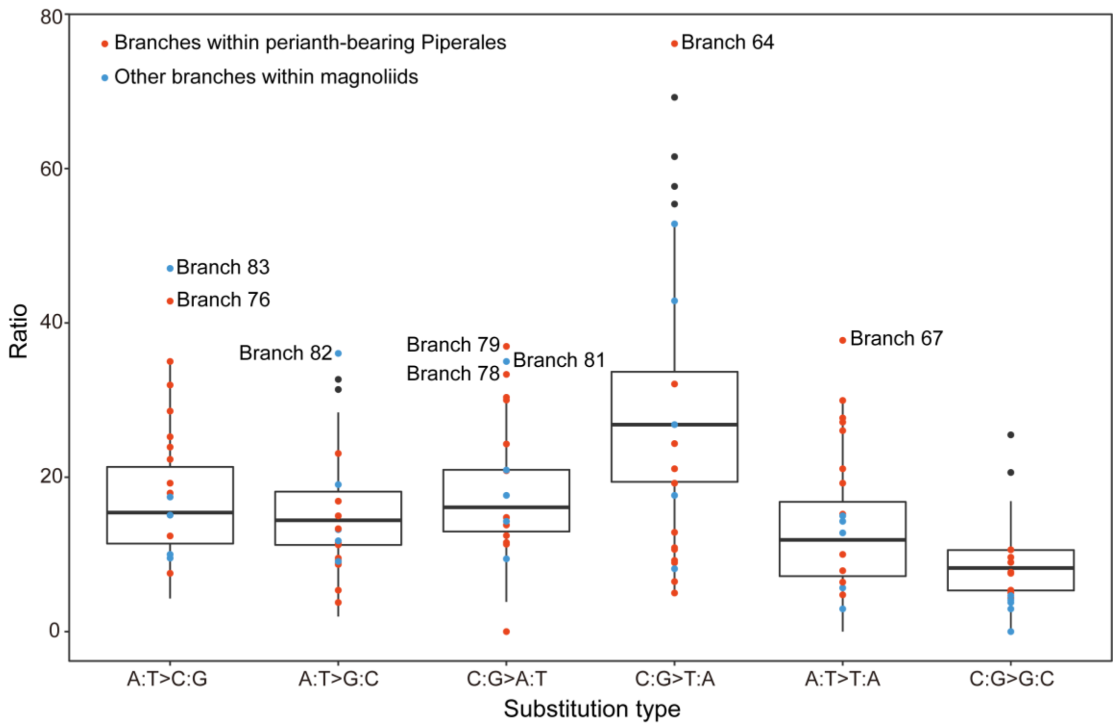

Fig. S6 Frequencies of different substitution types among angiosperm mitochondrial protein-coding genes. Black vertical line: median; box: upper and lower quartile, including 50% of the distribution; whiskers: minimum and maximum of the data, provided that their length does not exceed 1.5x the interquartile range; black circles: outliers. The data used to draw this figure can be found in Table S8.
